# MicroRNA‐Mediated Autophagy and Cardiovascular Diseases: Exploring the Crossroads of Atherosclerosis and Beyond

**DOI:** 10.1002/jcla.70310

**Published:** 2026-09-07

**Authors:** Sepehr Ebrahimi‐Dehkordi, Amir Hossein Barjasteh, Aida Zarei, Sina Bazmi, Sarah Vaseghi, Sara Ashna, Amirreza Solati, Ali Yousefzadeh, Bita Safarzadeh Nasrabad, Zahra Mohammadi, Hamed Haddad Kashani, Mohammad Hossein Pourhanifeh

**Affiliations:** ^1^ Student Research Committee Shahrekord University of Medical Sciences Shahrekord Iran; ^2^ Immunology Department, Faculty of Medicine Mashhad University of Medical Sciences Mashhad Iran; ^3^ Research Hub Institute Tehran Iran; ^4^ USERN Office Fasa University of Medical Sciences Fasa Iran; ^5^ Department of Radiology Tabriz University of Medical Sciences Tabriz Iran; ^6^ Student Research Committee Islamic Azad University Science and Research Branch Tehran Iran; ^7^ Student Research Committee Birjand University of Medical Sciences Birjand Iran; ^8^ Student Research Committee Tabriz University of Medical Sciences Tabriz Iran; ^9^ Student Research Committee, School of Medicine Ardabil University of Medical Sciences Ardabil Iran; ^10^ Anatomical Sciences Research Center, Institute for Basic Sciences Kashan University of Medical Sciences Kashan Iran; ^11^ Research Center for Biochemistry and Nutrition in Metabolic Diseases, Institute for Basic Sciences Kashan University of Medical Sciences Kashan Iran

**Keywords:** epithelial cell, non‐coding RNA, oxidized low density lipoprotein, programmed cell death, vascular aging, vascular aneurysm

## Abstract

**Background:**

Atherosclerosis (AS) is a complex cardiovascular disease characterized by the accumulation of lipids, inflammatory cells, and fibrous materials within arterial walls. Autophagy plays a critical role in maintaining cellular homeostasis and regulating lipid metabolism in vascular cells. MicroRNAs (miRNAs), small non‐coding RNA molecules involved in post‐transcriptional gene regulation, have emerged as key modulators of autophagy and contributors to AS progression.

**Methods:**

This review synthesizes current evidence on the interplay between miRNAs and autophagy in the context of AS and related cardiovascular diseases. A comprehensive literature search was conducted to identify studies investigating miRNA‐mediated regulation of autophagic flux, associated molecular pathways, and their implications for vascular health and disease.

**Results:**

Specific miRNAs have been identified as critical regulators of autophagy in AS, influencing autophagic flux through various signaling pathways. These miRNA‐mediated mechanisms contribute to lipid metabolism, inflammation, and cellular homeostasis in vascular cells, thereby affecting the initiation and progression of atherosclerotic lesions.

**Conclusion:**

Elucidating the regulatory networks linking miRNAs and autophagy provides valuable insights into novel therapeutic strategies targeting aberrant miRNA‐mediated autophagy. These findings highlight the potential of miRNAs as therapeutic and diagnostic targets for preventing or mitigating the development of AS and related cardiovascular diseases.

## Graphical abstract

This graphical abstract illustrates the dynamic bidirectional crosstalk between microRNA biogenesis and autophagy, which serves as a critical regulatory axis in cardiovascular homeostasis. We highlight how specific miRNAs modulate autophagic flux to mitigate vascular inflammation and plaque instability, while concurrently, the autophagic machinery regulates miRNA levels to govern endothelial survival and apoptosis.

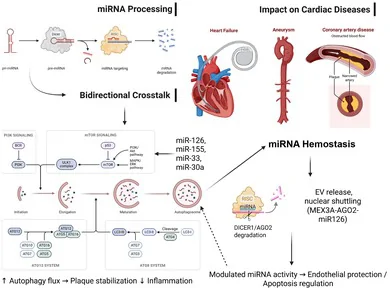

**Abbreviations**
AGEs: advanced glycation end‐products
AGO2: argonaute 2
Akt: protein kinase B
AMPK: AMP‐activated protein kinase
AS: atherosclerosis
ATG: autophagy‐related gene/protein
BECN1: Beclin‐1 gene
CaMKIIδ: calcium/calmodulin‐dependent protein kinase II delta
CVD: cardiovascular disease
DLK1: delta‐like 1 homolog
DNMT3A: DNA methyltransferase 3A
EC: endothelial cell
ECM: extracellular matrix
EPOR: erythropoietin receptor
ER: endoplasmic reticulum
FOXO4: forkhead box O4
HUVECs: human umbilical vein endothelial cells
IL‐1β: interleukin‐1 beta
LAMP2: lysosomal‐associated membrane protein 2
LC3: microtubule‐associated protein 1 light chain 3
MEX3A: Mex‐3 RNA binding family member A
Mfn2: mitofusin 2
MI: myocardial Infarction
mTOR: mammalian target of rapamycin
NDP52: nuclear dot protein 52 (CALCOCO2)
NF‐κB: nuclear factor kappa B
NLRP3: NLR family pyrin domain containing 3
ox‐LDL: oxidized low‐density lipoprotein
PDGF‐BB: platelet‐derived growth factor type BB
PI3K: phosphoinositide 3‐kinase
Rheb: Ras homolog enriched in brain
RISC: RNA‐induced silencing complex
RRAGD: Ras‐related GTP binding D
SQSTM1: sequestosome‐1
SREBP‐2: sterol regulatory element‐binding protein 2
ULK1: Unc‐51 like autophagy activating kinase 1
UVRAG: UV radiation resistance‐associated gene
VCAM1: vascular cell adhesion molecule 1
VSMCs: vascular smooth muscle cells
XBP‐1: X‐box binding protein 1
α‐SMA: alpha‐smooth muscle actin

## 1 Introduction

Atherosclerosis (AS) is one of the main causes of death worldwide despite significant therapeutic advances in cardiovascular diseases (CVD) [1, 2]. Since 2019, these entities have been responsible for approximately 18 million deaths annually, accounting for ~13 of global mortality [3]. As is determined by accumulating calcification, fibrous elements, and lipids in large and medium‐sized arteries. This procedure starts with activating the endothelium, which causes vessels to narrow and activate inflammatory pathways [4, 5]. Endothelial dysfunction, lipid peroxidation, and recruitment of infiltrating monocytes and vascular smooth muscle cells (VSMC) lead to arterial remodeling and progression of atherosclerotic disease [6, 7, 8]. Because the unpredictable atherosclerotic plaque can rupture at any moment, early diagnosis and treatment of atherosclerotic disease are essential [3]. Emerging evidence suggests that oxidized low‐density lipoprotein (ox‐LDL), metabolic stressors, and inflammation promote cell death type II or autophagy [9]. Autophagy delivers various cellular substances to lysosomes for degradation and recycling [10]. By these duties, autophagy helps scavenge damaged organelles and degraded proteins generated during atherosclerotic disease. Proper activation of autophagy associated with stress tolerance protects the cell by degrading damaged organelles, promoting cholesterol efflux, and degrading proteins in atherosclerotic plaques. However, excessive autophagic response can lead to the destruction of pro‐survival proteins and cell death. Thus, taking advantage of the protective effects of autophagy without triggering undesirable death pathways will be challenging [11, 12]. MicroRNAs (miRNAs) are short, non‐coding, single‐stranded RNAs that consist of 19–23 nucleotides in length that complement or bind to each other with a target gene messenger RNA (mRNA) to inhibit translation of the target mRNA or promote mRNA degradation [13]. By influencing protein translation, miRNAs are considered major regulators of biological processes such as cell death and autophagy [14, 15, 16]. MiRNAs play an essential role in regulating autophagy signaling cascades by modulating the expression of autophagy‐related genes [17, 18]. Recently, it has been demonstrated that autophagy regulation by miRNAs is involved in the pathogenesis of several cancers, including gastrointestinal cancers [19, 20], brain tumors [21], and gynecological cancers [22]. In this review of literature, we aimed to study miRNAs associated with autophagy regulation in AS and other cardiovascular disorders.

## 2 MiRNAs: Biogenesis and Roles in Cardiovascular Diseases

### 2.1 MiRNAs Biogenesis and Function

**FIGURE 1:**
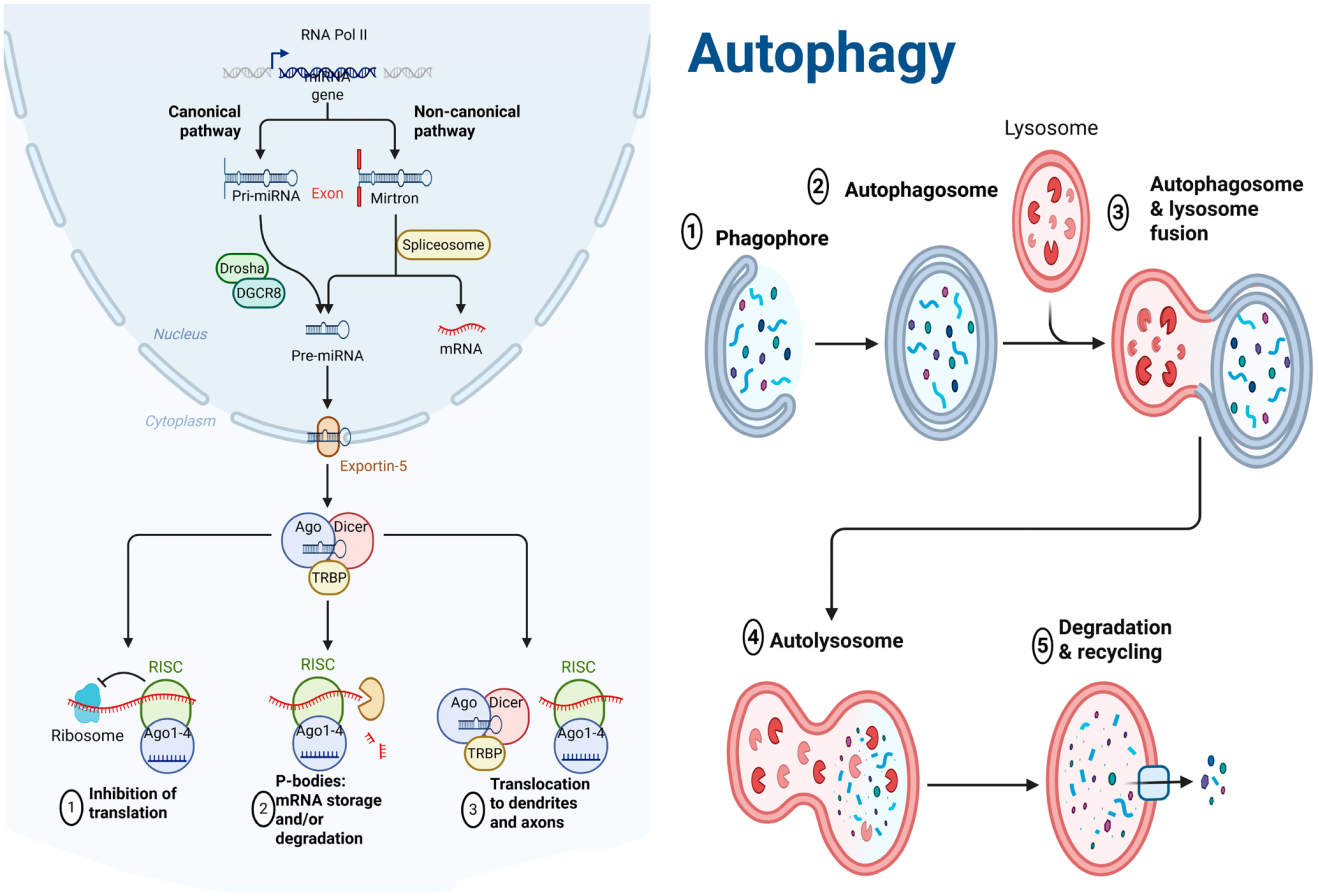
The illustration of miRNA biogenesis and autophagy mechanism.

The canonical biogenesis of microRNAs (miRNAs) begins with transcription of primary miRNAs (pri‐miRNAs) by RNA polymerase II or III. These pri‐miRNAs are cleaved by the Drosha‐DGCR8 complex in the nucleus to produce precursor miRNAs (pre‐miRNAs), which are then exported to the cytoplasm via Exportin‐5. In the cytoplasm, Dicer processes pre‐miRNAs into mature miRNA duplexes, followed by loading into the RNA‐induced silencing complex (RISC), where the mature miRNA guides post‐transcriptional repression of target mRNAs through degradation or translational inhibition. In addition to this well‐established pathway, non‐canonical biogenesis routes exist, including Drosha‐independent or Dicer‐independent maturation, as well as chemical modifications such as A‐to‐I editing, which alter miRNA stability, targeting specificity, or processing efficiency (Figure 1) [23]. For instance, editing can redirect miRNA targeting in vascular cells, contributing to endothelial dysfunction in AS. Moreover, mature miRNAs exhibit unconventional functions beyond cytoplasmic mRNA repression. In the nucleus, mature miRNAs translocate via Importin‐8 and regulate gene transcription by interacting with promoters or enhancers to activate or suppress expression through epigenetic modifications (histone acetylation or methylation) or by modulating non‐coding RNA (ncRNA) maturation, such as blocking pri‐miRNA cleavage [24, 25]. A notable example is miR‐126‐5p, which, in complex with AGO2 and MEX3A on autophagosomal surfaces, enters the nucleus to non‐canonically inhibit caspase‐3 dimerization, conferring anti‐apoptotic protection to endothelial cells in AS [24, 26]. Additionally, miRNAs can engage in RISC‐independent interactions, such as direct binding to proteins or mitochondrial roles in transcriptional control. In AS, small non‐coding RNAs like miRNAs can be glycosylated for cell surface exposure or released extracellularly via extracellular vesicles to act as ligands for receptors, influencing intercellular signaling and plaque progression. Understanding these atypical mechanisms expands the functional landscape of miRNAs and underscores their role in cardiovascular disease pathogenesis.

Additionally, miRNAs engage in RISC‐independent interactions, such as direct binding to proteins (biophysical regulation of enzyme activity) or mitochondrial roles in transcriptional control [27]. In AS, small ncRNAs like miRNAs can be glycosylated for cell surface exposure or released extracellularly via extracellular vesicles to act as ligands for receptors, influencing intercellular signaling, vascular inflammation, and plaque progression [28, 29]. These atypical localizations and mechanisms, including RISC heterogeneity, expand miRNA functionality and underscore their role in CVD pathogenesis, from endothelial integrity to fibrous cap stability. Understanding these deviations offers new avenues for therapeutic targeting in vascular disorders.

### 2.2 The Role of miRNAs in Cardiovascular Diseases

Increasing evidence indicates that miRNAs play a critical role in regulating cellular functions within the cardiovascular system [30, 31]. The miRNAs highlighted here were selected based on their representation of diverse pathogenic mechanisms in CVDs, with validation in clinical cohorts or in vivo models, though this is not exhaustive. Notably, several miRNAs exhibit vascular enrichment, high expression in endothelial cells (ECs), vascular smooth muscle cells (VSMCs), and macrophages, and are pivotal in AS progression. For instance, miR‐126, abundantly expressed in ECs, promotes vascular integrity by inhibiting leukocyte adhesion and enhancing endothelial repair, thereby attenuating early AS lesion formation [32, 33, 34]. Similarly, miR‐21, upregulated in response to vascular injury, drives VSMC proliferation and migration while promoting EC dysfunction, contributing to neointimal hyperplasia and plaque instability [35, 36]. miR‐155 modulates inflammatory responses in AS by polarizing macrophages toward a pro‐inflammatory M1 phenotype and suppressing cholesterol efflux, exacerbating foam cell formation [37]. miR‐33, clustered with SREBF2, inhibits ABCA1‐mediated reverse cholesterol transport in macrophages and hepatocytes, directly promoting lipid accumulation in plaques and representing a therapeutic target for HDL elevation [38].

Vascular‐enriched miRNA clusters further underscore this theme: the miR‐143/145 cluster maintains the contractile VSMC phenotype and inhibits atherogenic switching [39], while the miR‐17‐92 cluster regulates angiogenesis and EC survival in hypoxic vascular environments [40]. Comprehensive reviews detail these and other vascular miRNAs, miR‐92a in endothelial inflammation; miR‐34a in senescence, highlighting their diagnostic and therapeutic potential in AS and related CVDs [3, 27, 41, 42].

Since lipid accumulation is central to the development of AS, it is important to note that the progression of atherosclerotic plaques starts with endothelial activation and formation, followed by foam cell formation and monocyte recruitment [43, 44, 45]. Therefore, targeting the interaction between monocytes and endothelial cells may offer a promising strategy for preventing AS at its early stages [46]. VSMCs influence AS by forming the fibrous cap, stabilizing plaque, and accumulating lipids through internalizing ox‐LDL [6, 47]. Overexpressed miR‐125b‐1‐3p has been found to trigger mammalian target of the rapamycin (mTOR) signaling, diminishing the formation of foam cells in VSMCs and suppressing AS [48]. Given miRNA application as diagnostic tools for ventricular hypertrophy, Qi et al. identified miR‐204‐5p, miR‐26b‐5p, and miR‐497‐3p as biomarkers differentiating physiological from pathological ventricular hypertrophy [49]. In hypertensive individuals, elevated miR‐365 expression was a great prognostic marker for the development of left ventricular hypertrophy in the presence of high blood pressure [50]. Fu et al. discovered a negative correlation between increased miR‐26b and left ventricular diastolic function in hypertensive patients [51]. Additionally, miR‐301a‐3p, miR‐652‐3p, miR‐23b‐3p, miR‐374a‐5p, miR‐26b‐5p, miR‐423‐5p, miR‐483‐5p, and miR‐199a‐5p were found to be linked to severe left ventricular remodeling in the miRNA profile analysis [52]. Apart from analyzing miRNA involvement in the pathogenesis of hypertrophy‐associated hypertension, pulmonary arterial hypertension has been explored in miRNA investigations as well. In line with this idea, the miR‐429‐3p/Rac1 axis has been suggested as a remodeling regulator during pulmonary arterial hypertension [53]. Düzgün et al. investigated lower expression levels of miR‐143‐3p and miR‐21‐3p in patients with pulmonary hypertension. Furthermore, a substantial decrease was observed in miR‐143, miR‐138, miR‐145, miR‐204, miR‐206, miR‐190, and miR‐208 levels [54]. Heart failure, which mainly occurs as a consequence of other heart diseases like myocardial infarction (MI) or cardiomyopathy, causes detrimental effects on quality of life, long‐term hospitalization, and high mortality [55, 56, 57]. Some circulatory miRNAs might be detectable in the course of the disease, providing information about its progression and molecular mechanisms [58]. Vu et al. observed that patients with heart failure had upregulated levels of miR‐132 and miR‐152, which could serve as important diagnostic biomarkers [59]. An analysis of hospitalized post‐MI patients revealed that miR‐23a‐3p, miR‐221‐3p, and miR‐210‐3p were distinct indicators for cardiovascular mortality following an acute MI and long‐term hospitalizations for heart failure [60].

## 3 Autophagy and Vascular Biology

Autophagy is a cellular recycling system in eukaryotic cells where dysfunctional components are transported to the vacuole or lysosome for destruction (Figure 1). It begins with forming a membrane structure called a phagophore, mediated by the Golgi apparatus, endosomes, and the endoplasmic reticulum (ER) [61]. Three basic types of autophagy have been discovered: (A) autophagy induced by chaperone, in which a chaperone complex plus lysosomal membrane proteins decompose cytosolic proteins; (B) microautophagy performed by direct lysosomal uptake of cytoplasmic components; and (C) macroautophagy performed by a specified bilayer membrane named autophagosomes, which deliver cytoplasmic cargo to lysosomes directly. During this recycling process, transporters and lysosomal permeases export other by‐products and amino acids to the cytoplasm, where they can be reused for metabolism and the manufacturing of macromolecules [62]. Regarding its defensive role, autophagy behavior is a developmental strategy to protect organisms against pathophysiological triggers such as advanced glycation end products [63], hypoxia [64], and ox‐LDL in cardiomyocytes, myofibroblasts, VSMCs, and epithelial cells (EC) [65]. It has been illustrated that X‐box binding protein‐1 mRNA splicing in the endoplasmic reticulum and activating Beclin‐1 transcription are crucial steps for the initiation of autophagic response in ECs [66]. Numerous intracellular vascular disease‐related irritants, such as ox‐LDL and reactive species, in ECs and VSMCs, initiate autophagy as a pro‐survival mechanism [65]. Excess free cholesterol triggers autophagy in VSMCs, leading to improved formation of autophagic vacuoles and conversion of microtubule‐associated protein 1 light chain 3 (LC3‐I) to LC3‐II, maintaining VSMCs hemostasis by scavenging injured organelles [67]. However, an imperfect or exaggerated autophagy response is nearly always equates to cell damage, so that a defensive backup mechanism (mainly supported by nuclear factor erythroid 2–related factor 2 pathway) is required to maintain cell survival [68]. One of the main positive regulators of autophagy in the cardiovascular system is platelet‐derived growth factor‐BB (PDGF‐BB), which is secreted by different types of cells following an injury and triggers autophagy [69, 70]. Autophagy associated with PDGF‐BB aids in eliminating contractile proteins like proteins hurt by lipid electrophiles [71]. In cardiomyocytes, autophagic cell death mainly takes place through either uncontrolled cargo materials deprivation due to increased autophagic flux or extreme buildup of autophagosomes [72]. It should be said that specific nutrients or metabolic products, such as vitamin D, C6‐ceramide, and advanced glycation end‐products (AGEs), instigate the formation of autophagosomes through interaction with Beclin‐1 [73, 74, 75]. Moreover, the autophagic system in VSMCs can be a promising therapeutic target for inhibiting tumor progression because of its close interaction with capable functions of angiogenesis and neovascularization. Autophagy triggers vital angiogenesis‐related mediators that can be therapeutic targets to inhibit tumor progression [76]. Many documents that are still in progress direct that autophagy dysregulation can lead to pathogenic alterations in the function and structure of the cardiac muscle [72]. Mutation in the lysosomal‐associated membrane protein‐2 (LAMP2) gene, which causes autophagy faults, has been suggested to be linked to Danon disease, which is revealed by cardiac dysfunction and cardiomyopathy [77]. In fact, LAMP2 knockout mice demonstrate that the survived animals develop pathological conversions and numerous immature mortalities not only in the cardiovascular system but also in non‐muscular organs, such as the liver and the pancreas. Induction of cardiac hypertrophy using Angiotensin II has been accompanied by stimulated autophagy in the heart, upregulated levels of Beclin‐1 and ATG‐5, and increased LC3‐II/LC‐I ratio [78, 79]. Aliskiren (a renin inhibitor) reversed all these alternations in mice [80]. Recent research has thoroughly explored the role of autophagy in the development of AS. Autophagy plays a regulatory role in macrophage foam cells by facilitating the release of unbound cholesterol [81]. In addition to lacking pro‐autophagic gene ATG‐5 in lipid‐loaded macrophages, impaired regulation of mTOR signaling was detected in macrophage‐derived foam cells [82]. Therefore, autophagy helps cholesterol outflow from macrophage foam cells, simplifying the deterioration of atherosclerotic plaques. Also, the enhancement in oxidative stress and malfunctioning autophagy of macrophages leads to helping plaque necrosis through reduced phagocytic elimination of apoptotic macrophages. When oxidative stress occurs, autophagy plays a dual role. In the context of mild oxidative stress, autophagy effectively scavenges the ROS. However, in extreme oxidative stress, autophagy may not be able to compensate for the damage to mitochondria. In this situation, the intrinsic apoptotic pathway would be activated, and cytochrome C would leak from mitochondria [83]. Additionally, increased autophagy and the buildup of ROS encourage the development of ceroid (a complex mixture of proteins formed during lipid oxidation), which is found in advancing atherosclerotic plaques [84]. Altogether, autophagy has beneficial consequences on cell hemostasis but may also be implicated in the development of cardiovascular diseases and AS.

### 3.1 Bidirectional Crosstalk: Autophagy's Regulation of miRNA Function and Homeostasis in Vascular Biology

While miRNAs are well‐established regulators of autophagy, emerging evidence highlights a reciprocal relationship wherein autophagy modulates miRNA biogenesis, stability, trafficking, and even non‐canonical functions. This bidirectional interplay is essential for cellular homeostasis, particularly in vascular cells where it influences endothelial integrity, inflammatory responses, and atherosclerotic plaque stability. Autophagy can selectively degrade key components of the miRNA machinery, regulate the intracellular and extracellular trafficking of RNA‐binding proteins (RBPs) and ncRNAs, and influence the assembly of RNA‐induced silencing complexes (RISC) via pathways like mTORC signaling. Dysregulation of these processes contributes to vascular pathologies, including endothelial dysfunction and AS progression.

Autophagy selectively targets miRNA‐processing enzymes and effectors, such as DICER1 and AGO2, to fine‐tune miRNA stability and activity. In miRNA‐free states, DICER1 and AGO2 are degraded via selective autophagy mediated by receptors like NDP52 (CALCOCO2). This process establishes a checkpoint for miRNA loading into AGO2, ensuring proper miRNA homeostasis [85, 86]. Inhibition of autophagy via ATG5/ATG7 depletion leads to accumulation of DICER1 and AGO2, but paradoxically reduces miRNA levels and repressive activity over time, as shown by decreased miR‐16 and let‐7a abundance and impaired target repression RAS, HMGA2 [85, 86]. Similarly, autophagy degrades SQSTM1/p62, which in turn reduces DICER1 and AGO2 levels, elevating specific miRNAs like MIRLET7A‐3P to suppress cell motility, a mechanism observed in cancer but with implications for vascular smooth muscle cell (VSMC) migration in AS [87]. In vascular contexts, this degradation could prevent excessive miRNA dysregulation under stress; for example, ox‐LDL exposure, maintaining endothelial barrier integrity and reducing inflammatory cell infiltration into plaques.

The autophagy machinery also governs the trafficking of RBPs and ncRNAs, both intracellularly and extracellularly via extracellular vesicles (EVs). The LC3‐conjugation system, involving ATG7, ATG12, and ATG3 directs RBPs like HNRNPK and SAFB to EVs by lipidating LC3 on endosomal membranes, facilitating cargo loading and secretion [88]. This pathway enriches EVs with small ncRNAs such as snoRNAs, altering their extracellular composition in autophagy‐deficient cells. In vascular biology, EV‐mediated ncRNA trafficking is critical for intercellular communication; for instance, endothelial‐derived EVs carrying miRNAs modulate VSMC phenotype and macrophage polarization in atherosclerotic lesions [88]. Autophagy‐dependent nuclear trafficking of miRNAs further exemplifies this: under high‐shear stress or mTOR inhibition, autophagy promotes the assembly of a MEX3A‐AGO2‐miR‐126‐5p complex on autophagosomal surfaces, enabling nuclear import. In the nucleus, miR‐126‐5p dissociates from AGO2 and inhibits caspase‐3 (CASP3) dimerization non‐canonically, conferring endothelial protection against apoptosis and attenuating AS [89, 90]. This mechanism underscores autophagy's role in vascular integrity, as endothelial autophagy deficiency exacerbates plaque progression in vivo.

Additionally, the mTORC pathway, a central autophagy regulator, influences RISC assembly and miRNA function. mTOR activation enhances GW182 expression, promoting high‐molecular‐weight RISC (HMW‐RISC) formation and efficient miRNA‐mediated repression, independent of miRNA abundance change [91]. Conversely, autophagy induction via mTOR inhibition, can shift miRNA complexes toward low‐molecular‐weight forms or non‐canonical roles, as seen with MIR126‐5p [89]. In quiescent cells, AGO2‐bound miRNAs may reside in reservoirs not associated with mRNA, with mTOR/autophagy balancing their engagement [91]. Although some studies focus on transposon silencing by nuclear AGO2 [91], the autophagy‐mTOR axis likely integrates these functions in vascular cells, where mTOR hyperactivation in AS promotes inflammation and plaque instability.

Overall, autophagy's regulation of miRNA homeostasis extends beyond degradation to include trafficking and non‐canonical aptamer‐like interactions, with profound implications for AS. For example, autophagy‐mediated miRNA nuclear shuttling protects endothelium from ox‐LDL‐induced apoptosis, while EV secretion of ncRNAs may propagate anti‐inflammatory signals in plaques. Future studies targeting this crosstalk via NDP52 or MEX3A modulation could yield novel therapeutics for CVDs.

## 4 Autophagy‐Related miRNAs in Atherosclerosis

As AS evolves, VSMCs reside in pre‐atherosclerotic intima and invade media, expanding the initial lesion [92, 93]. Moreover, VSMCs participate in foam cell development, which had been believed to originate primarily from macrophages. Morphologically, VSMCs can take on diverse forms similar to foam cells and macrophages, displaying their versatility [94]. VSMCs are also required to construct a shielding fibrous cap loaded with extracellular matrix (ECM). This fibrous cap shelters the necrotic core of the plaque, stabilizing it and preventing rupture [92, 95]. This understanding of VSMC behavior is essential for discovering possible targets for therapeutic approaches in AS [6]. Autophagy activation in VSMCs has been detected in an array of vascular disorders [96]. Evidence suggests that miRNAs associated with autophagy impact vascular health and AS through their various epigenetic effects [97, 98]. In this regard, downregulation of miR‐100, the 14q32 miRNA cluster, miR‐92a, and miR‐15a‐16 [99, 100, 101] and increased expression of miR‐424, the miR‐106b‐25 cluster, miR‐26b, and miR‐132 [102, 103, 104, 105] are crucial in promoting vascularization. Moreover, miR‐503 downregulation in altered vessel permeability [106], overexpression of miR‐18a and miR‐19a in angiogenesis [107], miR‐126 upregulation in re‐endothelialization [108], miR‐143‐145 upregulation in atheroprotective [109], and miR‐10a upregulation in anti‐inflammation [110] have been documented. Regulating miRNAs to maintain an appropriate autophagy balance is crucial and essential for preserving VSMC function and preventing incorrect remodeling. Shi et al. demonstrated that miR‐30a blocked autophagy and enhanced contractile properties of aortic VSMCs by affecting the autophagy protein Beclin‐1 [96]. Beclin‐1, encoded by the BECN‐1 gene, is crucial for regulating membrane movement, tumor suppression, and autophagy in human cells. It forms part of the Class III PI3K complexes responsible for initiating autophagy and interacts with apoptosis‐related proteins like Bcl‐2 [111, 112]. Shi et al. showed that Myocardin, a transcription factor critical to preserving the contractile aortic VSMC phenotype, induces miR‐30a transcription and inhibits autophagy to reduce hyperplasia after carotid artery injury [96]. Besides controlling autophagy through contractile phenotype regulation, Zheng et al. [113] have observed that miR‐130a and autophagy‐related 2B (ATG‐2B) suppress VSMC proliferation. Hypoxia is one of the substantial stimulants for autophagy regulation, and it impacts the phenotypic aspects and function of VSMCs [114]. It has been found that miR‐210 accelerates autophagy and angiogenesis in schwannoma cells via interaction with hypoxia‐inducible Factor 1‐alpha and vascular endothelial growth factor [115].

### 4.1 MiRNAs Regulation in Beneficial Autophagy

Several studies have highlighted the beneficial effects of modulating autophagy through miRNAs in atherosclerotic endothelial cells. Research by Tang et al. demonstrated that miR‐126‐5p safeguards endothelial cells from damage in AS by restoring impaired autophagic flux, which is commonly observed in endothelial cells exposed to ox‐LDL. This protective effect is achieved through the inhibition of the PI3K/Akt/mTOR pathway, a critical regulator of autophagy. Their findings revealed that overexpression of miR‐126‐5p effectively reversed autophagy dysfunction and cellular damage caused by ox‐LDL [116]. Notably, both strands of miR‐126 contribute to vascular protection against AS: while miR‐126‐5p promotes endothelial proliferation by suppressing DLK1 and limits lesion formation at non‐predilection sites, miR‐126‐3p attenuates inflammation by downregulating VCAM1, thereby reducing leukocyte adhesion and early plaque development [117]. Interestingly, autophagy reciprocally regulates miR‐126 homeostasis; under high shear stress, autophagic vesicles facilitate the assembly of a Mex3a‐AGO2‐miR‐126‐5p complex, enabling nuclear import where miR‐126‐5p non‐canonically inhibits caspase‐3 dimerization to prevent apoptosis. Endothelial‐specific ablation of ATG5 or Mex3a disrupts this pathway, exacerbating AS in vivo [89]. This bidirectional loop underscores miR‐126‐5p's dual role in autophagy modulation and endothelial resilience, with implications for shear‐stress‐prone arterial regions.

Induced autophagy through miR‐126‐5p inhibition could also effectively eliminate damaged cells in the context of myocardial infarction [118]. In an atherosclerotic plaque environment, enhancing autophagic flux could be beneficial by reducing the burden of ox‐LDL‐filled cells. MiR‐155 has been shown to induce autophagy in human umbilical vein endothelial cells (HUVECs) stimulated by ox‐LDL by targeting the PI3K/Akt/mTOR pathway [119]. MiR‐155 can be highlighted as a therapeutic target for AS due to its direct attachment to the PI3K subunit and enhancing autophagy in ECs. Likewise, Lv et al. revealed a notable rise in miR‐155 expression induced by ox‐LDL, resulting in increased autophagic function. This effect was mediated through the suppression of Ras homolog enriched in the brain (Rheb), consequently inhibiting the mTOR/P70S6K/4EBP signaling pathway [120]. Wang et al. found that miR‐761 interfered with foam cell formation and reduced inflammatory cytokine production via autophagy regulation. Their study showed that miR‐761 inhibited lipid accumulation by modulating autophagy in macrophages through the mTOR/ULK1 signaling [121]. Zhu et al. observed that miR‐149 promoted autophagy by blocking the Akt/mTOR pathway, which lessens ox‐LDL‐induced EC damage. Indeed, overexpressing miR‐149 in human ECs decreased caspase‐3 activity and enhanced efficacious autophagy [122]. It is clear that the majority of the miRNAs discussed impact the Akt/mTOR pathway. The Akt/mTOR pathway is a crucial regulator of autophagy, influencing cell growth and metabolism [123, 124]. Under nutrient‐rich conditions, Akt expression leads to the phosphorylation of mTOR, which suppresses autophagy. However, when experiencing stress, reduced Akt activity suppresses mTOR and triggers autophagy [125, 126, 127]. Dysregulation of this system has been linked to diseases like cancer and neurological disorders, where inappropriate autophagic activity threatens cell survival [128]. MiR‐29a has been suggested to induce macrophage autophagy, preventing the expansion of atherosclerotic plaque. Shao et al. demonstrated that miR‐29a targets the PI3K/AKT/mTOR pathway, leading to increased autophagy and reduced plaque size [129]. Lipid uptake in VSMCs can be controlled by miRNA regulation. In keeping with this idea, overexpression of miR‐125b‐1‐3p has been shown to reduce atherosclerotic plaque formation through mTOR signaling and lessen lipid uptake in VSMCs in vivo [48]. Furthermore, inhibition of pyroptosis via NLRP3 inflammasome suppression was observed following miR‐99a‐5p induction and macrophage autophagy promotion [130]. Altering lipoprotein metabolism can also be a therapeutic focus for AS prevention and treatment. For example, studies demonstrate that miR‐222 can indirectly reduce VLDL production [131, 132].

### 4.2 MiRNAs That Inhibit Protective Autophagy and Thereby Promote Atherosclerosis

In contrast to miRNAs that enhance protective autophagy, several miRNAs suppress autophagic flux in vascular cells and macrophages, thereby impairing lipid clearance, cholesterol efflux, and resolution of inflammation [133, 134]. Consequences that accelerate atherosclerotic plaque progression. In this context, autophagy remains fundamentally protective, and its inhibition is pro‐atherogenic.

MiR‐129‐5p is upregulated in endothelial cells of high‐fat‐diet ApoE^−^/^−^ mice and directly targets BECN1, reducing Beclin‐1 protein levels, impairing autophagosome formation, and promoting endothelial dysfunction and lesion development [131]. Similarly, miR‐384‐5p is elevated in macrophages of atherosclerotic mice and suppresses Beclin‐1, leading to defective autophagy, accumulation of lipid droplets, and enhanced foam‐cell formation [132]. MiR‐33 (both 33a and 33b) inhibits autophagic flux in macrophages indirectly by blocking lysosomal acid lipase trafficking and by sustaining lipid overload, resulting in defective cholesterol homeostasis and impaired efferocytosis of apoptotic cells [135]. MiR‐214‐3p directly targets ATG5 in endothelial cells, reducing LC3‐II levels and autophagosome maturation under ox‐LDL stress, which exacerbates endothelial injury and monocyte adhesion [136]. Likewise, miR‐216a and miR‐483‐5p impair autophagic flux in ox‐LDL‐treated HUVECs by downregulating Beclin‐1 and other core machinery components, leading to increased apoptosis and reduced cell viability [38, 137]. In macrophages, miR‐135b‐5p represses the erythropoietin receptor, activates PI3K/Akt signaling, suppresses autophagy, and promotes apoptotic cell death [95]. In VSMCs, miR‐130a inhibits proliferation and migration in part by targeting ATG2B and reducing the LC3‐II/LC3‐I ratio, although the net effect on plaque stability remains context‐dependent [111].

While excessive, uncontrolled autophagy can become detrimental in very late‐stage plaques or under extreme oxidative stress by triggering autophagic cell death, no miRNAs have yet been definitively shown to drive such pathologically hyperactive autophagy in AS. Thus, the vast majority of pro‐atherogenic miRNAs act by blocking protective autophagy rather than by inducing harmful over‐activation. Table 1 summarizes the current data on the significant roles of autophagy‐associated miRNAs in the pathogenesis of AS. Understanding these fundamental mechanisms contributes to designing further investigations as well as developing more effective therapeutic approaches.

**TABLE 1:** MIRNAs involved in autophagy regulation in atherosclerosis studies.

	MiRNA	MiRNA regulation	Effect on autophagy	Target	Study outcomes	Model	References
1	MiR‐126	Upregulation	Activator	PI3K/Akt/mTOR pathway	MiR‐126 overexpression can reverse ox‐LDL‐induced injury in HUVECs^a^	In vitro	[116]
2	MiR‐33	Downregulation	Activator	ATG5, lysosomal genes via SREBP‐2 axis	MiR‐33 regulates AS via efferocytosis MiR‐33 reduces cholesterol mobilization through a lysosomal‐dependent mechanism The relationship between miR‐33 and SREBP‐2 help cholesterol balance in cells	In vivo	[136]
3	MiR‐214‐3p	Upregulation	Inhibitor	ATG5 (3′UTR binding)	MiR‐214‐3p overexpression reduced cell viability and increased apoptosis in HUVECs MiR‐214‐3p encouraged THP‐1 monocyte adhesion	In vitro	[138]
4	MiR‐30a	Upregulation	Inhibitor	Beclin‐1 (BECN1)	MiR‐30a‐5p regulates the phenotypic switch of VSMCs by interfering with autophagy MiR‐30a family reduces balloon‐injured rat carotid arteries MiR‐30a‐5p stabilized contractile proteins of VSMCs^a^ following PDGF‐BB^a^ treatment	In vitro; In vivo	[96]
5	MiR‐130a	Downregulation	Inhibitor	ATG2B	MiR‐130a is downregulated in arteriosclerosis obliterans of small to medium‐sized lower extremity arteries MiR‐130a suppresses VSMCs proliferation, invasion, and migration	In vivo	[113]
6	MiR‐155	Upregulation	Activator	PI3K/Akt/mTOR pathway (via PI3K, Rheb)	MiR‐155 regulates endothelial cell apoptosis and VSMC migration MiR‐155 downregulates the PI3K/Akt/mTOR signaling via targeting PI3K and Rheb	In vitro	[139]
7	MiR‐155	Upregulation	Activator	Rheb/mTOR pathway	MiR‐155 triggers autophagosome accumulation in EA.hy926 endothelial cells MiR‐155 triggers autophagy through inhibition of Rheb/mTOR	In vitro	[120]
8	MiR‐155	Upregulation	Activator	PI3K/Akt/mTOR pathway	MiR‐155 increases autophagosomes and autolysosome numbers MiR‐155 downregulates p62/GAPDH ratio	In vitro	[140]
9	MiR‐761	Downregulation	Inhibitor	mTOR/ULK1 signaling	MiR‐761 represses foam cell formation and reduces atherogenic inflammatory cytokines IL‐1β and IL‐18 MiR‐761 enhances autophagy through mTOR/ULK1 signaling	In vitro	[121]
10	MiR‐149	Upregulation	Activator	Akt/mTOR pathway	MiR‐149 has a protective effect on endothelial cells through ox‐LDL MiR‐149 downregulates the expression of Akt, p‐Akt, mTOR, and p‐mTOR in ox‐LDL‐treated HUVECs.	In vitro	[122]
11	MiR‐99a	Downregulation	Inhibitor	mTOR	MiR‐99a‐5p inhibits NLRP3 inflammasome activation and inflammatory cytokines MiR‐99a‐5p overexpression inhibits mTOR expression	In vivo	[130]
12	MiR‐145‐5p	Downregulation	Inhibitor	CaMKIIδ → AMPK/mTOR/ULK1 axis	MiR‐145‐5p inhibits CaMKIIδ expression CaMKIIδ activates the AMPK/mTOR/ULK1 pathway in human aortic VSMCs	In vitro	[141]
13	MiR‐125b‐1‐3p	Downregulation	Activator	RRAGD/mTOR/ULK1 pathway	MiR‐125b‐1‐3p overexpression decreases lipid uptake and deposition in VSMCs MiR‐125b‐1‐3p inhibiting foam cell formation in VSMCs through RRAGD/mTOR/ULK1	In vitro	[48]
14	MiR‐129‐5p	Upregulation	Inhibitor	Beclin‐1	MiR‐129‐5p decreases Beclin‐1 expression in endothelial cells	In vivo; In vitro	[135]
15	MiR‐384‐5p	Upregulation	Inhibitor	Beclin‐1 (3′UTR binding)	A high‐fat diet induces upregulation of miR‐384‐5p MiR‐384‐5p upregulation decreases Beclin‐1 in macrophages via 3′‐UTR binding	In vivo	[142]
16	MiR‐520a‐3p	Upregulation	Inhibitor	UVRAG	MiR‐520a‐3p inhibits macrophage polarization Inhibition of miR‐520a‐3p increases the expression of α‐SMA and collagen MiR‐520a‐3p decreases M1 and increases M2 macrophage polarization markers expression markers	In vitro	[143]
17	MiR‐512‐3p	Upregulation	Inhibitor	XBP‐1 (spliced/unspliced ratio)	MiR‐512‐3p downregulation enhances cell viability and suppresses cell apoptosis in ox‐LDL‐treated HUVECs MiR‐512‐3p downregulation directly targets XBP‐1	In vitro; In vivo	[144]
18	MiR‐216a	Upregulation	Inhibitor	ATG5, Beclin‐1	MiR‐216a overexpression represses ATG5 and BECN1 expression in young HUVECs MiR‐216a motivates ox‐LDL accumulation and monocyte adhesion in HUVECs	In vitro	[145]
19	MiR‐135b	Upregulation	Inhibitor	EPOR (erythropoietin receptor)	MiR‐135 activates the PI3K/Akt pathway MiR‐135b inhibition mitigates inflammation and atherosclerotic plaque formation	In vivo	[146]
20	MiR‐328‐3p	Downregulation	Activator	FOXO4	MiR‐328‐3p downregulation has a protective effect on HUVECs against ox‐LDL MiR‐328‐3p targets FOXO4 affecting VSMCs, bone marrow‐derived cells, or the expression of NF‐kB‐activated inflammatory cytokines	In vitro	[147]
21	MiR‐483‐5p	Upregulation	Inhibitor	Akt/mTOR pathway	MiR‐483‐5p protects HUVECs from ox‐LDL‐induced injury MiR‐149 inhibits the Akt/mTOR pathway	In vitro	[148]

## 5 The Role of Autophagy‐Related miRNAs in Other CVDs

### 5.1 Cardiac Fibrosis

It has been described that miRNA regulation might interfere with fibroblast migration and fibrosis formation facilities. Sun et al. indicated that miR‐19a‐3p/19b‐3p potentially suppressed epithelial‐mesenchymal transition, the production of the ECM, and the invasive capabilities of human cardiac fibroblasts by acting on TGF‐β RII [149]. These findings recommended that miR‐19a‐3p/19b‐3p has an anti‐fibroblast effect by its regulation of cardiac autophagy [150]. These findings recommended that miR‐19a‐3p/19b‐3p has an anti‐fibroblast effect by regulating cardiac autophagy. Zhao et al. demonstrated that DNA methyltransferase 3A (DNMT3A) plays a crucial role in the regulation of miR‐200b, which subsequently influences cardiac fibroblast autophagy during cardiac fibrosis [151]. The activity of DNMT3A and DNMT3B at cytosines is crucial for genome regulation and development [152]. Studies indicate that inactivating DNMT3A in macrophages enhances their interaction with cardiac fibroblasts, leading to increased cardiac fibrosis [153]. Yang et al. revealed that miRNA‐122‐5p plays a crucial role in regulating apoptosis and autophagy in rat cardiac fibrosis via the Apelin/AMPK/mTOR pathway [154].

### 5.2 Cardiac Hypertrophy

The heart responds to sustained elevations in blood pressure or volume by enlarging its muscle mass; a condition generally referred to as cardiac hypertrophy [155, 156]. Dysregulated cardiac autophagy is a key feature of a diseased heart, with a growing recognition of the link between autophagy and cardiac hypertrophy. The miR302–367 cluster was reported as a myocardial autophagy regulator which supported hypertrophy through PI3K/AKT/mTORC1 signaling [157]. Emerging evidence indicates that several miRNAs, including miR‐26b‐5p, miR‐497‐3p, and miR‐204‐5p, play a role in regulating autophagy‐related genes through the modulation of the miRNAs‐autophagy axis. This regulatory mechanism may provide insights into differentiating between physiological and pathological forms of LVH [49]. Since miRNAs are crucial in cardiac hypertrophy, Xu X and colleagues conducted a study showing that miRNA‐17‐5p suppresses the mitochondrial fusion protein Mitofusin 2 (Mfn2). This inhibition activates the phosphatidylinositol‐3‐kinase (PI3K)/AKT/mTOR pathway and inhibits autophagy, ultimately expanding cardiac hypertrophy [158]. The treatment with Isoproterenol, a non‐selective beta‐adrenergic agonist, led to considerable hypertrophy and a notable increase in the expression of miRNA‐146b‐5p in cultured neonatal rat cardiomyocytes as well as in the hearts of mice, as conducted by Liu et al. [159]. Conversely, miRNA‐146b‐5p inhibition reversed cardiac hypertrophy caused by Isoproterenol by preventing autophagy depression.

### 5.3 Vascular Calcification

Vascular calcification is the deposition of hydroxyapatite in vascular walls, contributing to severe cardiovascular events in individuals with AS promoter factors like hypertension, diabetes, and renal disease [160, 161]. It was found that the interaction of miR‐320a‐3p with vitamin D regulated the formation of calcification in vasculature. These miRNAs enhanced autophagy and reduced calcification in VSMCs through silencing programmed cell death 4 gene [162]. Research has shown that miR‐30b plays a key role in autophagy caused by hyperphosphatemia by regulating Beclin‐1. MiR‐30b has been found to protect VSMCs against calcification formation by enhancing autophagy and mitochondrial function through SOX9 and the mTOR pathway suppression [163]. The progression of vascular calcification in diabetes is closely linked to the suppression of autophagy in VSMCs. Cao et al. identified increased miRNA‐32‐5p in macrophages of individuals with elevated glucose levels [164].

### 5.4 Vascular Aneurysms

MiRNAs can participate in aortic integrity regulation by affecting the proliferation, apoptosis, differentiation, and functions of VSMCs in large vessels. While miRNA‐29 [165], miRNA‐205 [166], miRNA‐195 [165], and miRNA‐21 [167] were found to be upregulated, miRNA‐26a [168] and miRNA‐143/145 [169] were downregulated in aortic aneurysms. The miR‐29 family reduces the gene expression responsible for encoding ECM proteins, including Collagen types I and III, Elastin, and Fibrillin 1. A murine model study by Boon et al. indicated that increased miR‐29 levels boosted ECM degradation and increased the risk of aortic aneurysm development with aging compared to young animals. Decreased plasma levels of miRNA‐195 as a potent regulator of the aortic ECM were observed in a mice model of aortic aneurysm [165]. Overexpression of miR‐21 causes aortic aneurysm development through elevated phosphorylation and activation of AKT. It was revealed that smoking is the most important modifiable risk factor for aortic aneurysm development, and upregulation of miR‐21 in smoker patients with aortic aneurysm was associated with nicotine administration [170]. Furthermore, overexpression of TGF‐β‐associated miRNAs like miR‐26a was observed in aneurysmal compared with non‐aneurysmal tissue [171]. Evidence has uncovered miRNA regulation in intracranial aneurysm pathogenesis. As Wang et al. have described, dominant expression of miR‐29a was detected in intracranial aneurysm patients compared to the control group [172]. Zhao et al. study showed miRNA‐29a reduction decreased brain VSMC apoptosis, suggesting involvement in intracranial aneurysm progression by controlling mitochondrial apoptosis [173]. Serum levels of miR‐513b‐5p and IL‐6 were markedly decreased in the intracranial aneurysm patients in both types of ruptured and un‐ruptured intracranial aneurysms [174]. It was found that high miR‐126 expression in plasma was linked to unruptured type aneurysms [175, 176]. Likewise, lesion size and miR‐126 expression were introduced as predictable indices and independent risk factors for rupture types [176].

## 6 MiRNAs Associated With Vascular Senescence and Aging

**FIGURE 2:**
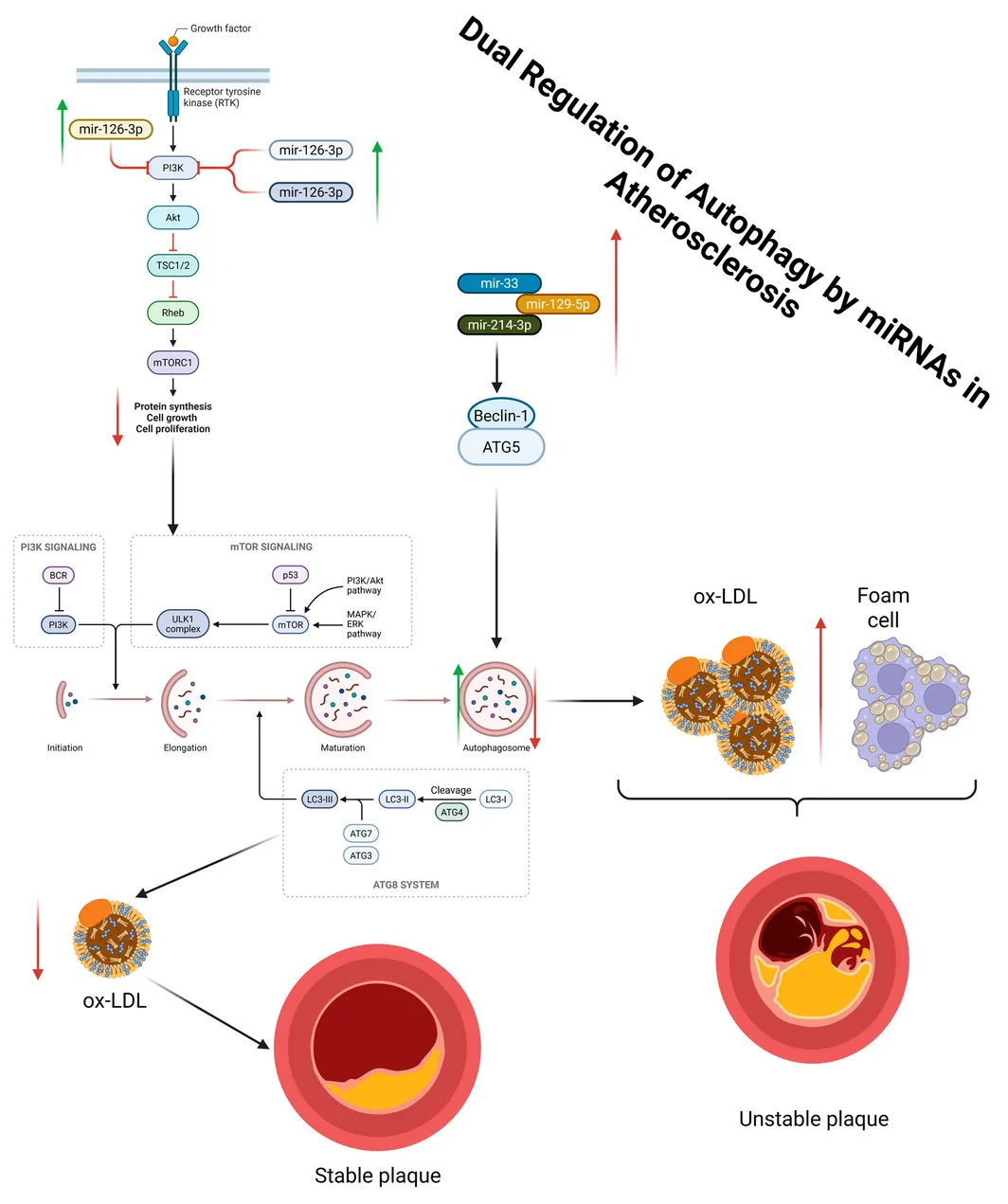
Schematic presentation of underlying mechanisms of autophagy‐related miRNAs in atherosclerosis.

Vascular aging involves changes in arterial wall structure and function with age, leading to increased stiffness, decreased elasticity, higher pulse wave velocity, and systolic pressure. This entity induces chronic arterial inflammation and remodeling through VSMC migration and proliferation, change in ECM components, and epigenetic features [177, 178]. Studies in individuals over 60 y/o have shown that circulating miR‐181a‐5p, miR‐1248, and miR‐151a‐3p were downregulated with age [179]. Autophagy interacts with mitochondria and telomerase to establish cardiovascular homeostasis [180]. Age‐related miRNA regulation has been detected in age‐related cardiac pathologies compared to normal‐aged hearts. Zhang et al. revealed that the expression of miR‐21, miR‐22, and the miR‐17–92 cluster is meaningfully altered in normal‐aged hearts with aging [181]. Highly expressed MiR‐21 in the aged heart or in post‐MI condition promotes the transition of cardiac fibroblasts to myofibroblasts by repressing Jagged1 and Smad7, leading to fibrotic tissue formation [182, 183]. One of the age‐related phenotypic changes in cardiomyocytes is lessening the contractile function, which is mediated by miR‐34a interaction with protein phosphatase PNUTS and DNA damage response [184]. MiR‐18a and miR‐19a/b have been reported to accelerate collagen synthesis and fibrotic remodeling via interaction with pro‐fibrotic CTGF and TSP1 genes [185]. The senescence of EC plays an important role in the vascular aging process. Exosomal miR‐214 has been reported as an inhibitor of the ataxia telangiectasia mutated factor in EC, causing EC senescence and stimulating migration and angiogenesis [186]. Figure 2 illustrates the dual role of miRNAs and autophagy in CVDs.

## 7 Conclusion

The intricate relationship between autophagy regulation and miRNAs indicates a potential therapeutic approach for AS and cardiovascular conditions. AS is a significant contributor to mortality worldwide, highlighting the urgent necessity for innovative treatment approaches. Autophagy emerges as a significant player in the pathogenesis of atherosclerotic plaques due to its dual purpose of preserving cellular homeostasis and triggering cell death. Apparently, miRNAs control autophagy‐related functions by interacting with cellular death, inflammation, and lipid metabolism. The multifaceted impacts of miRNAs on AS include alterations in endothelial function, foam cell production, plaque stability, and vascular remodeling. Dysregulation of particular miRNAs can worsen or improve AS progression, indicating their potential as therapeutic targets. Furthermore, autophagy plays a role in other vascular pathology beyond AS, including heart failure/fibrosis, cardiomyopathy, hypertension, and hypertrophy. Understanding the interaction between miRNAs and autophagy processes may provide a practical guide for developing targeted therapies that alter these signalings to reduce AS progression and enhance cardiovascular outcomes. Future research should further explore the specific miRNA‐autophagy links, discovering their precise role in disease etiology and developing novel therapeutic strategies.

## Author Contributions

**Sepehr Ebrahimi‐Dehkordi:** conceptualization, methodology, investigation, writing original draft. **Aida Zarei: writing, review and editing, methodology.** **Sina Bazmi:** data curation, visualization, writing, review and editing. **Sarah Vaseghi:** methodology, investigation, data curation, writing original draft. **Sara Ashna:** validation, writing, review and editing. **Amirreza Solati:** investigation, resources, data curation, writing original draft. **Ali Yousefzadeh:** methodology, validation, visualization, writing, review and editing. **Bita Safarzadeh Nasrabad:** investigation, data curation, project administration, writing, review and editing. **Zahra Mohammadi:** writing, review, and editing. **Hamed Haddad Kashani:** conceptualization, supervision, review and editing. **Mohammad Hossein Pourhanifeh:** conceptualization, methodology, supervision, writing original draft, writing review and editing. **Amir Hossein Barjasteh:** investigation, resources, project administration, writing review and editing. All authors read and approved the final manuscript.

## Funding

The authors have nothing to report.

## Disclosure

AI Declaration: AI was only used to paraphrase the text and check for any errors in English writing. Then the data was overseen by the authors and verified. No generative AI was used in writing the manuscript.

## Ethics Statement

The study is formed as a narrative review and doesn't need ethical approval.

## Consent

All participants provided consent for the publication of anonymized data and findings from this study. No identifiable personal information is included in the publication.

## Conflicts of Interest

The authors declare no conflicts of interest.

## Data Availability

The authors have nothing to report.

## References

[1] G. Ghorbannezhad , S. Mehrabadi , N. Golampour‐Shamkani , et al., “Genetic Determinants of Response to Statins in Cardiovascular Diseases,” Current Cardiology Reviews 20, no. 2 (2024): 20–28. 38204221 10.2174/011573403X267793231220114042 PMC11107471

[2] I. Hetherington and H. Totary‐Jain , “Anti‐atherosclerotic therapies: milestones, challenges, and emerging innovations,” Molecular Therapy 30, no. 10 (2022): 3106–3117. 36065464 10.1016/j.ymthe.2022.08.024 PMC9552812

[3] Z. Li , Y. Zhao , S. Suguro , and R. Suguro , “MicroRNAs Regulate Function in Atherosclerosis and Clinical Implications,” Oxidative Medicine and Cellular Longevity 2023, no. 1 (2023): 2561509. 37675243 10.1155/2023/2561509 PMC10480027

[4] S. Jebari‐Benslaiman , U. Galicia‐García , A. Larrea‐Sebal , et al., “Pathophysiology of Atherosclerosis,” International Journal of Molecular Sciences 23, no. 6 (2022): 3346. 35328769 10.3390/ijms23063346 PMC8954705

[5] Y. Han , S. Liu , B. O. Ochieng , et al., “Multiscale Mechanical Study of Proanthocyanidins for Recovering Residual Stress in Decellularized Blood Vessels,” Advanced Healthcare Materials 14, no. 7 (2025): e2402250. 39801220 10.1002/adhm.202402250

[6] M. R. Bennett , S. Sinha , and G. K. Owens , “Vascular Smooth Muscle Cells in Atherosclerosis,” Circulation Research 118, no. 4 (2016): 692–702. 26892967 10.1161/CIRCRESAHA.115.306361 PMC4762053

[7] P. H. Wirtz and R. J. C. von Känel , “Psychological Stress, Inflammation, and Coronary Heart Disease,” Current Cardiology Reports 19 (2017): 1–10. 28932967 10.1007/s11886-017-0919-x

[8] W. Z. Wang , C. Liu , J. Q. Luo , et al., “A Novel Small‐Molecule PCSK9 Inhibitor E28362 Ameliorates Hyperlipidemia and Atherosclerosis,” Acta Pharmacologica Sinica 45, no. 10 (2024): 2119–2133. 38811775 10.1038/s41401-024-01305-9 PMC11420243

[9] Y. Osonoi , T. Mita , K. Azuma , et al., “Defective Autophagy in Vascular Smooth Muscle Cells Enhances Cell Death and Atherosclerosis,” Autophagy 14, no. 11 (2018): 1991–2006. 30025494 10.1080/15548627.2018.1501132 PMC6152523

[10] J. Debnath , N. Gammoh , and K. M. Ryan , “Autophagy and Autophagy‐Related Pathways in Cancer,” Nature Reviews. Molecular Cell Biology 24, no. 8 (2023): 560–575. 36864290 10.1038/s41580-023-00585-z PMC9980873

[11] D. Denton and S. J. C. D. Kumar , “Differentiation. Autophagy‐Dependent Cell Death,” Cell Death and Differentiation 26, no. 4 (2019): 605–616. 30568239 10.1038/s41418-018-0252-y PMC6460387

[12] L. Lin , M.‐X. Zhang , L. Zhang , et al., “Autophagy, Pyroptosis, and Ferroptosis: New Regulatory Mechanisms for Atherosclerosis,” Frontiers in Cell and Developmental Biology 9 (2022): 809955. 10.3389/fcell.2021.809955 PMC8793783 35096837

[13] M. Palizkaran Yazdi , A. Barjasteh , and M. Moghbeli , “MicroRNAs as the Pivotal Regulators of Temozolomide Resistance in Glioblastoma,” Molecular Brain 17, no. 1 (2024): 42. 38956588 10.1186/s13041-024-01113-6 PMC11218189

[14] E. F. Finnegan and A. E. Pasquinelli , “MicroRNA Biogenesis: Regulating the Regulators,” Critical Reviews in Biochemistry and Molecular Biology 48, no. 1 (2013): 51–68. 23163351 10.3109/10409238.2012.738643 PMC3557704

[15] J. S. Nahand , S. Taghizadeh‐boroujeni , M. Karimzadeh , et al., “microRNAs: New Prognostic, Diagnostic, and Therapeutic Biomarkers in Cervical Cancer,” Journal of Cellular Physiology 234, no. 10 (2019): 17064–17099. 30891784 10.1002/jcp.28457

[16] J. Sadri Nahand , M. Moghoofei , A. Salmaninejad , et al., “Pathogenic Role of Exosomes and microRNAs in HPV‐Mediated Inflammation and Cervical Cancer: A Review,” International Journal of Cancer 146, no. 2 (2020): 305–320. 31566705 10.1002/ijc.32688 PMC6999596

[17] L. B. Frankel and A. H. Lund , “MicroRNA Regulation of Autophagy,” Carcinogenesis 33, no. 11 (2012): 2018–2025. 22902544 10.1093/carcin/bgs266

[18] A. Fazlollahpour‐Naghibi , S. R. Taha , M. Shariatzadeh , et al., “Regulatory Effect of Non‐Coding RNAs on Programmed Cell Death in Melanoma: Novel Therapeutic Crosstalk for Targeted Therapy,” Current Cancer Drug Targets, 10.2174/0115680096329727250129173510.10. 40017243

[19] M. H. Pourhanifeh , M. Vosough , M. Mahjoubin‐Tehran , et al., “Autophagy‐Related microRNAs: Possible Regulatory Roles and Therapeutic Potential in and Gastrointestinal Cancers,” Pharmacological Research 161 (2020): 105133. 32822869 10.1016/j.phrs.2020.105133

[20] S. R. Taha , M. Karimi , B. Mahdavi , et al., “Crosstalk Between Non‐Coding RNAs and Programmed Cell Death in Colorectal Cancer: Implications for Targeted Therapy,” Epigenetics & Chromatin 18, no. 1 (2025): 3. 39810224 10.1186/s13072-024-00560-8 PMC11734566

[21] M. H. Pourhanifeh , M. Mahjoubin‐Tehran , M. R. Karimzadeh , et al., “Autophagy in Cancers Including Brain Tumors: Role of MicroRNAs,” Cell Communication and Signaling: CCS 18, no. 1 (2020): 88. 32517694 10.1186/s12964-020-00587-w PMC7285723

[22] M. Mobinikhaledi , A. Faridzadeh , T. Farkhondeh , M. H. Pourhanifeh , and S. Samarghandian , “The Roles of Autophagy‐Related miRNAs in Gynecologic Tumors: A Review of Current Knowledge for Possible Targeted Therapy,” Current Molecular Medicine 24, no. 10 (2024): 1269–1281. 39300715 10.2174/0115665240263059231002093454

[23] M. P. Dragomir , E. Knutsen , and G. A. Calin , “Classical and Noncanonical Functions of miRNAs in Cancers,” Trends in Genetics 38, no. 4 (2022): 379–394. 34728089 10.1016/j.tig.2021.10.002

[24] J. Gu , Y. Li , Y. Tian , Y. Zhang , Y. Cheng , and Y. Tang , “Noncanonical Functions of microRNAs in the Nucleus,” Acta Biochimica et Biophysica Sinica 56, no. 2 (2024): 151–161. 38167929 10.3724/abbs.2023268 PMC10984876

[25] W. Li , S. Wang , J. Xu , and J. Xiang , “Inferring Latent MicroRNA‐Disease Associations on a Gene‐Mediated Tripartite Heterogeneous Multiplexing Network,” IEEE/ACM Transactions on Computational Biology and Bioinformatics 19, no. 6 (2022): 3190–3201. 35041612 10.1109/TCBB.2022.3143770

[26] S. Zhou , S. Wang , Q. Wu , R. Azim , and W. Li , “Predicting Potential miRNA‐Disease Associations by Combining Gradient Boosting Decision Tree With Logistic Regression,” Computational Biology and Chemistry 85 (2020): 107200. 32058946 10.1016/j.compbiolchem.2020.107200

[27] D. Santovito and C. Weber , “Non‐Canonical Features of microRNAs: Paradigms Emerging From Cardiovascular Disease,” Nature Reviews. Cardiology 19, no. 9 (2022): 620–638. 35304600 10.1038/s41569-022-00680-2

[28] F. M. Farina , C. Weber , and D. Santovito , “The Emerging Landscape of Non‐Conventional RNA Functions in Atherosclerosis,” Atherosclerosis 374 (2023): 74–86. 36725418 10.1016/j.atherosclerosis.2023.01.009

[29] Z. Zhou , L. Zhuo , X. Fu , J. Lv , Q. Zou , and R. Qi , “Joint Masking and Self‐Supervised Strategies for Inferring Small Molecule‐miRNA Associations,” Molecular Therapy—Nucleic Acids 35, no. 1 (2024): 102103. 38261851 10.1016/j.omtn.2023.102103 PMC10794920

[30] M. H. Bao , G. Y. Li , X. S. Huang , L. Tang , L. P. Dong , and J. M. Li , “Long Noncoding RNA LINC00657 Acting as a miR‐590‐3p Sponge to Facilitate Low Concentration Oxidized Low‐Density Lipoprotein‐Induced Angiogenesis,” Molecular Pharmacology 93, no. 4 (2018): 368–375. 29436491 10.1124/mol.117.110650

[31] R. Kamvar , A. H. Barjasteh , M. Omouri‐Kharashtomi , et al., “The Crosstalk Between MicroRNAs and Sirtuins: A Bit Unexpected Therapeutic Target for Cardiovascular Diseases,” European Journal of Medical Research 30, no. 1 (2025): 969. 41088315 10.1186/s40001-025-03235-6 PMC12522758

[32] J. E. Fish , M. M. Santoro , S. U. Morton , et al., “miR‐126 Regulates Angiogenic Signaling and Vascular Integrity,” Developmental Cell 15, no. 2 (2008): 272–284. 18694566 10.1016/j.devcel.2008.07.008 PMC2604134

[33] L. Liao , Y. Tang , Y. Zhou , X. Meng , B. Li , and X. Zhang , “MicroRNA‐126 (MiR‐126): Key Roles in Related Diseases,” Journal of Physiology and Biochemistry 80, no. 2 (2024): 277–286. 38517589 10.1007/s13105-024-01017-y

[34] C. van Solingen , R. Bijkerk , H. C. de Boer , T. J. Rabelink , and A. J. van Zonneveld , “The Role of microRNA‐126 in Vascular Homeostasis,” Current Vascular Pharmacology 13, no. 3 (2015): 341–351. 23713864 10.2174/15701611113119990017

[35] R. Ji , Y. Cheng , J. Yue , et al., “MicroRNA Expression Signature and Antisense‐Mediated Depletion Reveal an Essential Role of MicroRNA in Vascular Neointimal Lesion Formation,” Circulation Research 100, no. 11 (2007): 1579–1588. 17478730 10.1161/CIRCRESAHA.106.141986

[36] C. Sabatel , L. Malvaux , N. Bovy , et al., “MicroRNA‐21 Exhibits Antiangiogenic Function by Targeting RhoB Expression in Endothelial Cells,” PLoS One 6, no. 2 (2011): e16979. 21347332 10.1371/journal.pone.0016979 PMC3037403

[37] R. L. Zhang , W. M. Wang , J. Q. Li , et al., “The Role of miR‐155 in Cardiovascular Diseases: Potential Diagnostic and Therapeutic Targets,” International Journal of Cardiology: Cardiovascular Risk and Prevention 24 (2025): 200355. 39760132 10.1016/j.ijcrp.2024.200355 PMC11699627

[38] K. J. Rayner , C. C. Esau , F. N. Hussain , et al., “Inhibition of miR‐33a/b in Non‐Human Primates Raises Plasma HDL and Lowers VLDL Triglycerides,” Nature 478, no. 7369 (2011): 404–407. 22012398 10.1038/nature10486 PMC3235584

[39] T. Boettger , N. Beetz , S. Kostin , et al., “Acquisition of the Contractile Phenotype by Murine Arterial Smooth Muscle Cells Depends on the Mir143/145 Gene Cluster,” Journal of Clinical Investigation 119, no. 9 (2009): 2634–2647. 19690389 10.1172/JCI38864 PMC2735940

[40] C. Doebele , A. Bonauer , A. Fischer , et al., “Members of the microRNA‐17‐92 Cluster Exhibit a Cell‐Intrinsic Antiangiogenic Function in Endothelial Cells,” Blood 115, no. 23 (2010): 4944–4950. 20299512 10.1182/blood-2010-01-264812

[41] L. J. F. Peters , E. A. L. Biessen , M. Hohl , C. Weber , E. P. C. van der Vorst , and D. Santovito , “Small Things Matter: Relevance of MicroRNAs in Cardiovascular Disease,” Frontiers in Physiology 11 (2020): 793. 32733281 10.3389/fphys.2020.00793 PMC7358539

[42] H. Zhou , W. Tang , J. Yang , J. Peng , J. Guo , and C. Fan , “MicroRNA‐Related Strategies to Improve Cardiac Function in Heart Failure,” Frontiers in Cardiovascular Medicine 8 (2021): 773083. 34869689 10.3389/fcvm.2021.773083 PMC8639862

[43] Q. Xiang , Y. Chen , X. Cheng , et al., “Non‐Targeted Metabolomics Reveals the Potential Role of MFSD8 in Metabolism in Human Endothelial Cells,” Molecular Biotechnology 68, no. 2 (2026): 631–643. 39992484 10.1007/s12033-025-01396-7

[44] C. L. Galindo , S. Khan , X. Zhang , Y. S. Yeh , Z. Liu , and B. Razani , “Lipid‐Laden Foam Cells in the Pathology of Atherosclerosis: Shedding Light on New Therapeutic Targets,” Expert Opinion on Therapeutic Targets 27, no. 12 (2023): 1231–1245. 38009300 10.1080/14728222.2023.2288272 PMC10843715

[45] L. Guo , K. Zhu , Y. N. Zhong , et al., “Structural Basis and Biased Signaling of Proton Sensation by GPCRs Mediated by Extracellular Histidine Rearrangement,” Molecular Cell 85, no. 8 (2025): 1658–1673.e1657. 40215959 10.1016/j.molcel.2025.03.018

[46] J. L. M. Björkegren and A. J. Lusis , “Atherosclerosis: Recent Developments,” Cell 185, no. 10 (2022): 1630–1645. 35504280 10.1016/j.cell.2022.04.004 PMC9119695

[47] H. Kang , G. Yan , X. Lin , et al., “The Substructure of the Endothelial Glycocalyx in Rat Aorta and Its Interaction With the Low‐Density Lipoproteins,” American Journal of Pathology 195, no. 10 (2025): 1936–1958. 40645580 10.1016/j.ajpath.2025.06.005 PMC12597546

[48] X. Chen , Y. Cao , Y. Guo , et al., “microRNA‐125b‐1‐3p Mediates Autophagy via the RRAGD/mTOR/ULK1 Signaling Pathway and Mitigates Atherosclerosis Progression,” Cellular Signalling 118 (2024): 111136. 38471617 10.1016/j.cellsig.2024.111136

[49] J. Qi , X. Luo , Z. Ma , B. Zhang , S. Li , and J. Zhang , “Downregulation of miR‐26b‐5p, miR‐204‐5p, and miR‐497‐3p Expression Facilitates Exercise‐Induced Physiological Cardiac Hypertrophy by Augmenting Autophagy in Rats,” Frontiers in Genetics 11 (2020): 78. 32140172 10.3389/fgene.2020.00078 PMC7042403

[50] H. B. Wu , C. S. Yang , Y. C. Wang , et al., “The Expression of miR‐365 in Serum of Hypertension Patients With Left Ventricular Hypertrophy Was Up‐Regulated, Which Was Positively Correlated With Left Ventricular Mass Index,” Pharmacogenomics and Personalized Medicine 14 (2021): 905–913. 34321907 10.2147/PGPM.S319945 PMC8312326

[51] J. Fu , F. Lin , Z. Pan , and C. Wu , “Correlations of Circulating miR‐26b Level With Left Ventricular Hypertrophy and Cardiac Function in Elderly Patients With Hypertension,” Pakistan Journal of Medical Sciences 37, no. 4 (2021): 966–971. 34290767 10.12669/pjms.37.4.4048 PMC8281164

[52] F. Eyyupkoca , K. Ercan , E. Kiziltunc , I. B. Ugurlu , A. Kocak , and N. Eyerci , “Determination of microRNAs Associated With Adverse Left Ventricular Remodeling After Myocardial Infarction,” Molecular and Cellular Biochemistry 477, no. 3 (2022): 781–791. 35048282 10.1007/s11010-021-04330-y

[53] R. Qi , Y. Zhang , and F. Yan , “Exosomes Enriched by miR‐429‐3p Derived From ITGB1 Modified Telocytes Alleviates Hypoxia‐Induced Pulmonary Arterial Hypertension Through Regulating Rac1 Expression,” Cell Biology and Toxicology 40, no. 1 (2024): 32. 38767703 10.1007/s10565-024-09879-0 PMC11106170

[54] Z. Düzgün , L. M. Kayıkçıoğlu , Ç. Aktan , et al., “Decreased Circulating microRNA‐21 and microRNA‐143 Are Associated to Pulmonary Hypertension,” Turkish Journal of Medical Sciences 53, no. 1 (2023): 130–141. 36945942 10.55730/1300-0144.5566 PMC10388131

[55] B. Ibanez , S. James , S. Agewall , et al., “2017 ESC Guidelines for the Management of Acute Myocardial Infarction in Patients Presenting With ST‐Segment Elevation: The Task Force for the Management of Acute Myocardial Infarction in Patients Presenting With ST‐Segment Elevation of the European Society of Cardiology (ESC),” European Heart Journal 39, no. 2 (2018): 119–177. 28886621 10.1093/eurheartj/ehx393

[56] M. Al‐Hemairy , M. A. Serhani , Y. Atif , and S. Amin , “Classification of Pervasive Healthcare Systems,” (2013), Paper presented at: 2013 Sixth International Conference on Developments in eSystems Engineering.

[57] S. Ahmed , A. Hilal‐Alnaqbi , M. Al Hemairy , and M. Al Ahmad , “ECG Abnormality Detection Algorithm,” International Journal of Advanced Computer Science and Applications 9 (2018): 215–219.

[58] C. D. Searles , “MicroRNAs and Cardiovascular Disease Risk,” Current Cardiology Reports 26, no. 2 (2024): 51–60. 38206553 10.1007/s11886-023-02014-1 PMC10844442

[59] D. M. Vu , A. P. Huynh , N. N. Q. Nguyen , et al., “Upregulation of Serum miR‐132 and miR‐152 in Vietnamese Patients With Heart Failure,” Malaysian Journal of Medical Sciences 31, no. 4 (2024): 91–100. 10.21315/mjms2024.31.4.7 PMC11377001 39247117

[60] L. M. Rincón , M. Rodríguez‐Serrano , E. Conde , et al., “Serum microRNAs Are Key Predictors of Long‐Term Heart Failure and Cardiovascular Death After Myocardial Infarction,” ESC Heart Failure 9, no. 5 (2022): 3367–3379. 35837763 10.1002/ehf2.13919 PMC9715833

[61] S. T. Majeed , R. Majeed , and K. I. Andrabi , “Expanding the View of the Molecular Mechanisms of Autophagy Pathway,” Journal of Cellular Physiology 237, no. 8 (2022): 3257–3277. 35791448 10.1002/jcp.30819

[62] W. Li , P. He , Y. Huang , et al., “Selective Autophagy of Intracellular Organelles: Recent Research Advances,” Theranostics 11, no. 1 (2021): 222–256. 33391472 10.7150/thno.49860 PMC7681076

[63] G. Zhao , X. Zhang , H. Wang , and Z. Chen , “Beta Carotene Protects H9c2 Cardiomyocytes From Advanced Glycation End Product‐Induced Endoplasmic Reticulum Stress, Apoptosis, and Autophagy via the PI3K/Akt/mTOR Signaling Pathway,” Annals of Translational Medicine 8, no. 10 (2020): 647. 32566584 10.21037/atm-20-3768 PMC7290636

[64] M. Deng , W. Zhang , L. Yuan , J. Tan , and Z. Chen , “HIF‐1a Regulates Hypoxia‐Induced Autophagy via Translocation of ANKRD37 in Colon Cancer,” Experimental Cell Research 395, no. 1 (2020): 112175. 32679233 10.1016/j.yexcr.2020.112175

[65] Y. L. Zhang , Y. J. Cao , X. Zhang , et al., “The Autophagy‐Lysosome Pathway: A Novel Mechanism Involved in the Processing of Oxidized LDL in Human Vascular Endothelial Cells,” Biochemical and Biophysical Research Communications 394, no. 2 (2010): 377–382. 20223224 10.1016/j.bbrc.2010.03.026

[66] S. Kelaini , R. Caines , L. Zeng , and A. Margariti , “X‐Box‐Binding Protein 1 Splicing Induces an Autophagic Response in Endothelial Cells: Molecular Mechanisms in ECs and Atherosclerosis,” in Autophagy: Cancer, Other Pathologies, Inflammation, Immunity, Infection, and Aging (Elsevier, 2017), 259–268.

[67] K. Xu , Y. Yang , M. Yan , J. Zhan , X. Fu , and X. Zheng , “Autophagy Plays a Protective Role in Free Cholesterol Overload‐Induced Death of Smooth Muscle Cells,” Journal of Lipid Research 51, no. 9 (2010): 2581–2590. 20484746 10.1194/jlr.M005702 PMC2918441

[68] R. M. da Costa , D. Rodrigues , C. A. Pereira , et al., “Nrf2 as a Potential Mediator of Cardiovascular Risk in Metabolic Diseases,” Frontiers in Pharmacology 10 (2019): 382. 31031630 10.3389/fphar.2019.00382 PMC6473049

[69] J. H. Han , H. S. Park , D. H. Lee , J. H. Jo , K. S. Heo , and C. S. Myung , “Regulation of Autophagy by Controlling Erk1/2 and mTOR for Platelet‐Derived Growth Factor‐BB‐Mediated Vascular Smooth Muscle Cell Phenotype Shift,” Life Sciences 267 (2021): 118978. 33412209 10.1016/j.lfs.2020.118978

[70] W. Liu , G. Li , J. Shi , et al., “NR4A1 Acts as a Novel Regulator of Platelet Activation and Thrombus Formation,” Circulation Research 136, no. 8 (2025): 809–826. 40035146 10.1161/CIRCRESAHA.124.325645 PMC11984555

[71] J. Li , L. Zhao , T. Yang , Y. J. Zeng , and K. Yang , “C‐Ski Inhibits Autophagy of Vascular Smooth Muscle Cells Induced by oxLDL and PDGF,” PLoS One 9, no. 6 (2014): e98902. 24887307 10.1371/journal.pone.0098902 PMC4041777

[72] S. Ikeda , D. Zablocki , and J. Sadoshima , “The Role of Autophagy in Death of Cardiomyocytes,” Journal of Molecular and Cellular Cardiology 165 (2022): 1–8. 34919896 10.1016/j.yjmcc.2021.12.006 PMC8940676

[73] V. Meyer , B. R. Jones , and L. Bornman , “Autophagy Efficacy and Vitamin D Status: Population Effects,” Cellular Immunology 352 (2020): 104082. 32241530 10.1016/j.cellimm.2020.104082

[74] Y. Xie , S. J. You , Y. L. Zhang , et al., “Protective Role of Autophagy in AGE‐Induced Early Injury of Human Vascular Endothelial Cells,” Molecular Medicine Reports 4, no. 3 (2011): 459–464. 21468592 10.3892/mmr.2011.460

[75] J. Alizadeh , S. C. da Silva Rosa , X. Weng , et al., “Ceramides and Ceramide Synthases in Cancer: Focus on Apoptosis and Autophagy,” European Journal of Cell Biology 102, no. 3 (2023): 151337. 37392580 10.1016/j.ejcb.2023.151337

[76] A. A. Kraya , S. Piao , X. Xu , et al., “Identification of Secreted Proteins That Reflect Autophagy Dynamics Within Tumor Cells,” Autophagy 11, no. 1 (2015): 60–74. 25484078 10.4161/15548627.2014.984273 PMC4502670

[77] Y. Zhai , J. Miao , Y. Peng , Y. Wang , J. Dong , and X. Zhao , “Clinical Features of Danon Disease and Insights Gained From LAMP‐2 Deficiency Models,” Trends in Cardiovascular Medicine 33, no. 2 (2023): 81–89. 34737089 10.1016/j.tcm.2021.10.012

[78] S. Liu , S. Chen , M. Li , et al., “Autophagy Activation Attenuates Angiotensin II‐Induced Cardiac Fibrosis,” Archives of Biochemistry and Biophysics 590 (2016): 37–47. 26562437 10.1016/j.abb.2015.11.001

[79] W. Zhao , Y. Li , L. Jia , L. Pan , H. Li , and J. Du , “Atg5 Deficiency‐Mediated Mitophagy Aggravates Cardiac Inflammation and Injury in Response to Angiotensin II,” Free Radical Biology & Medicine 69 (2014): 108–115. 24418158 10.1016/j.freeradbiomed.2014.01.002

[80] L. Q. Weng , W. B. Zhang , Y. Ye , et al., “Aliskiren Ameliorates Pressure Overload‐Induced Heart Hypertrophy and Fibrosis in Mice,” Acta Pharmacologica Sinica 35, no. 8 (2014): 1005–1014. 24998254 10.1038/aps.2014.45 PMC4125714

[81] M. Ouimet , V. Franklin , E. Mak , X. Liao , I. Tabas , and Y. L. Marcel , “Autophagy Regulates Cholesterol Efflux From Macrophage Foam Cells via Lysosomal Acid Lipase,” Cell Metabolism 13, no. 6 (2011): 655–667. 21641547 10.1016/j.cmet.2011.03.023 PMC3257518

[82] X. Wang , L. Li , X. Niu , et al., “mTOR Enhances Foam Cell Formation by Suppressing the Autophagy Pathway,” DNA and Cell Biology 33, no. 4 (2014): 198–204. 24512183 10.1089/dna.2013.2164 PMC3967384

[83] S. Bolisetty and E. A. Jaimes , “Mitochondria and Reactive Oxygen Species: Physiology and Pathophysiology,” International Journal of Molecular Sciences 14, no. 3 (2013): 6306–6344. 23528859 10.3390/ijms14036306 PMC3634422

[84] C. Carresi , R. Mollace , R. Macrì , et al., “Oxidative Stress Triggers Defective Autophagy in Endothelial Cells: Role in Atherothrombosis Development,” Antioxidants 10, no. 3 (2021): 387. 33807637 10.3390/antiox10030387 PMC8001288

[85] D. Gibbings , S. Mostowy , F. Jay , Y. Schwab , P. Cossart , and O. Voinnet , “Selective Autophagy Degrades DICER and AGO2 and Regulates miRNA Activity,” Nature Cell Biology 14, no. 12 (2012): 1314–1321. 23143396 10.1038/ncb2611 PMC3771578

[86] D. Gibbings , S. Mostowy , F. Jay , Y. Schwab , P. Cossart , and O. Voinnet , “Corrigendum: Selective Autophagy Degrades DICER and AGO2 and Regulates miRNA Activity,” Nature Cell Biology 17, no. 8 (2015): 1088. 10.1038/ncb3208 26239531

[87] C. C. Liao , M. Y. Ho , S. M. Liang , and C. M. Liang , “Autophagic Degradation of SQSTM1 Inhibits Ovarian Cancer Motility by Decreasing DICER1 and AGO2 to Induce MIRLET7A‐3P,” Autophagy 14, no. 12 (2018): 2065–2082. 30081720 10.1080/15548627.2018.1501135 PMC6984764

[88] A. M. Leidal , H. H. Huang , T. Marsh , et al., “The LC3‐Conjugation Machinery Specifies the Loading of RNA‐Binding Proteins Into Extracellular Vesicles,” Nature Cell Biology 22, no. 2 (2020): 187–199. 31932738 10.1038/s41556-019-0450-y PMC7007875

[89] D. Santovito , V. Egea , K. Bidzhekov , et al., “Noncanonical Inhibition of Caspase‐3 by a Nuclear microRNA Confers Endothelial Protection by Autophagy in Atherosclerosis,” Science Translational Medicine 12, no. 546 (2020): eaaz2294. 32493793 10.1126/scitranslmed.aaz2294

[90] D. Santovito , V. Egea , K. Bidzhekov , et al., “Autophagy Unleashes Noncanonical microRNA Functions,” Autophagy 16, no. 12 (2020): 2294–2296. 33054575 10.1080/15548627.2020.1830523 PMC7751630

[91] G. La Rocca , S. H. Olejniczak , A. J. González , et al., “In Vivo, Argonaute‐Bound microRNAs Exist Predominantly in a Reservoir of Low Molecular Weight Complexes Not Associated With mRNA,” Proceedings of the National Academy of Sciences of the United States of America 112, no. 3 (2015): 767–772. 25568082 10.1073/pnas.1424217112 PMC4311832

[92] M. O. J. Grootaert and M. R. Bennett , “Vascular Smooth Muscle Cells in Atherosclerosis: Time for a Re‐Assessment,” Cardiovascular Research 117, no. 11 (2021): 2326–2339. 33576407 10.1093/cvr/cvab046 PMC8479803

[93] E. El Shebiny , S. Shoeib , S. M. Moeen , et al., “Serum Hepcidin: An Atherosclerotic Biomarker in Rheumatoid Arthritis Patients: A Multicenter Case‐Control Study,” Eurasian Journal of Medicine and Oncology 8, no. 2 (2024): 165–172.

[94] G. A. Francis , “The Greatly Under‐Represented Role of Smooth Muscle Cells in Atherosclerosis,” Current Atherosclerosis Reports 25, no. 10 (2023): 741–749. 37665492 10.1007/s11883-023-01145-8 PMC10564813

[95] L. Chen , Z. Jiang , L. Yang , et al., “HPDA/Zn as a CREB Inhibitor for Ultrasound Imaging and Stabilization of Atherosclerosis Plaque,” Chinese Journal of Chemistry 41, no. 2 (2023): 199–206.

[96] D. Shi , J. Ding , S. Xie , et al., “Myocardin/microRNA‐30a/Beclin1 Signaling Controls the Phenotypic Modulation of Vascular Smooth Muscle Cells by Regulating Autophagy,” Cell Death and Disease 13, no. 2 (2022): 121. 35136037 10.1038/s41419-022-04588-0 PMC8827084

[97] O. Woźniak , B. Mierzejewski , and E. Brzoska , “MicroRNA‐126: A Key Regulator of Angiogenesis, Inflammation, and Tumorigenesis—Exploring Its Multifaceted Functions in Vascular Health and Cancer,” Biochimica et Biophysica Acta, Molecular Basis of Disease 1871, no. 8 (2025): 167984. 40651584 10.1016/j.bbadis.2025.167984

[98] J. Zhang , Y. Chen , Y. Zhong , et al., “Intermittent Fasting and Cardiovascular Health: A Circadian Rhythm‐Based Approach,” Science Bulletin 70, no. 14 (2025): 2377–2389. 40480884 10.1016/j.scib.2025.05.017

[99] M. Besnier , S. Shantikumar , M. Anwar , et al., “miR‐15a/−16 Inhibit Angiogenesis by Targeting the Tie2 Coding Sequence: Therapeutic Potential of a miR‐15a/16 Decoy System in Limb Ischemia,” Molecular Therapy—Nucleic Acids 17 (2019): 49–62. 31220779 10.1016/j.omtn.2019.05.002 PMC6586592

[100] C. Zhai , J. Cheng , H. Mujahid , et al., “Selective Inhibition of PI3K/Akt/mTOR Signaling Pathway Regulates Autophagy of Macrophage and Vulnerability of Atherosclerotic Plaque,” PLoS One 9, no. 3 (2014): e90563. 24599185 10.1371/journal.pone.0090563 PMC3944201

[101] D. Mellis and A. Caporali , “MicroRNA Regulation of Vascular Function,” Vascular Biology 1, no. 1 (2019): H41–h46. 32923952 10.1530/VB-19-0009 PMC7439840

[102] G. Ghosh , I. V. Subramanian , N. Adhikari , et al., “Hypoxia‐Induced microRNA‐424 Expression in Human Endothelial Cells Regulates HIF‐α Isoforms and Promotes Angiogenesis,” Journal of Clinical Investigation 120, no. 11 (2010): 4141–4154. 20972335 10.1172/JCI42980 PMC2964978

[103] D. Mehlich , F. Garbicz , and P. K. Włodarski , “The Emerging Roles of the Polycistronic miR‐106b∼25 Cluster in Cancer—A Comprehensive Review,” Biomedicine & Pharmacotherapy = Biomedecine & Pharmacotherapie 107 (2018): 1183–1195. 30257332 10.1016/j.biopha.2018.08.097

[104] A. Martello , D. Mellis , M. Meloni , et al., “Phenotypic miRNA Screen Identifies miR‐26b to Promote the Growth and Survival of Endothelial Cells,” Molecular Therapy—Nucleic Acids 13 (2018): 29–43. 30227275 10.1016/j.omtn.2018.08.006 PMC6141730

[105] K. Xu , C. Chen , Y. Wu , M. Wu , and L. Lin , “Advances in miR‐132‐Based Biomarker and Therapeutic Potential in the Cardiovascular System,” Frontiers in Pharmacology 12 (2021): 751487. 34795586 10.3389/fphar.2021.751487 PMC8594750

[106] Y. He , Y. Cai , P. M. Pai , X. Ren , and Z. Xia , “The Causes and Consequences of miR‐503 Dysregulation and Its Impact on Cardiovascular Disease and Cancer,” Frontiers in Pharmacology 12 (2021): 629611. 33762949 10.3389/fphar.2021.629611 PMC7982518

[107] X. Jiang , W. L. Wooderchak‐Donahue , J. McDonald , et al., “Inactivating Mutations in Drosha Mediate Vascular Abnormalities Similar to Hereditary Hemorrhagic Telangiectasia,” Science Signaling 11, no. 513 (2018): eaan6831. 29339534 10.1126/scisignal.aan6831 PMC5811261

[108] Q. Qu , W. Bing , X. Meng , et al., “Upregulation of miR‐126‐3p Promotes Human Saphenous Vein Endothelial Cell Proliferation In Vitro and Prevents Vein Graft Neointimal Formation Ex Vivo and In Vivo,” Oncotarget 8, no. 63 (2017): 106790–106806. 29290989 10.18632/oncotarget.22365 PMC5739774

[109] W. Zhao , S. P. Zhao , and Y. H. Zhao , “MicroRNA‐143/−145 in Cardiovascular Diseases,” BioMed Research International 2015 (2015): 531740. 26221598 10.1155/2015/531740 PMC4499377

[110] A. Tahamtan , M. Teymoori‐Rad , B. Nakstad , and V. Salimi , “Anti‐Inflammatory MicroRNAs and Their Potential for Inflammatory Diseases Treatment,” Frontiers in Immunology 9 (2018): 1377. 29988529 10.3389/fimmu.2018.01377 PMC6026627

[111] R. Kang , H. J. Zeh , M. T. Lotze , and D. Tang , “The Beclin 1 Network Regulates Autophagy and Apoptosis,” Cell Death and Differentiation 18, no. 4 (2011): 571–580. 21311563 10.1038/cdd.2010.191 PMC3131912

[112] S. Tran , W. D. Fairlie , and E. F. Lee , “BECLIN1: Protein Structure, Function and Regulation,” Cells 10, no. 6 (2021): 1522. 34204202 10.3390/cells10061522 PMC8235419

[113] L. Zheng , Z. Wang , Z. Li , M. Wang , W. Wang , and G. Chang , “MicroRNA‐130a Inhibits Proliferation of Vascular Smooth Muscle Cells by Suppressing Autophagy via ATG2B,” Journal of Cellular and Molecular Medicine 25, no. 8 (2021): 3829–3839. 33611856 10.1111/jcmm.16305 PMC8051697

[114] H. Ren , R. Dai , W. N. Nik Nabil , Z. Xi , F. Wang , and H. Xu , “Unveiling the Dual Role of Autophagy in Vascular Remodelling and Its Related Diseases,” Biomedicine & Pharmacotherapy = Biomedecine & Pharmacotherapie 168 (2023): 115643. 37839111 10.1016/j.biopha.2023.115643

[115] T. Y. Wu , Q. Leng , and L. Q. Tian , “The microRNA‐210/Casp8ap2 Axis Alleviates Hypoxia‐Induced Myocardial Injury by Regulating Apoptosis and Autophagy,” Cytogenetic and Genome Research 161, no. 3–4 (2021): 132–142. 33882492 10.1159/000512254

[116] F. Tang and T. L. Yang , “MicroRNA‐126 Alleviates Endothelial Cells Injury in Atherosclerosis by Restoring Autophagic Flux via Inhibiting of PI3K/Akt/mTOR Pathway,” Biochemical and Biophysical Research Communications 495, no. 1 (2018): 1482–1489. 29203244 10.1016/j.bbrc.2017.12.001

[117] A. Schober , M. Nazari‐Jahantigh , Y. Wei , et al., “MicroRNA‐126‐5p Promotes Endothelial Proliferation and Limits Atherosclerosis by Suppressing Dlk1,” Nature Medicine 20, no. 4 (2014): 368–376. 10.1038/nm.3487 PMC4398028 24584117

[118] C.‐C. Shi , L. Y. Pan , Z. Y. Peng , and J. G. Li , “MiR‐126 Regulated Myocardial Autophagy on Myocardial Infarction,” European Review for Medical & Pharmacological Sciences 24, no. 12 (2020): 6971–6979. 32633391 10.26355/eurrev_202006_21689

[119] S. Yin , S. Yang , X. Pan , et al., “MicroRNA‐155 Promotes Ox‐LDL‐Induced Autophagy in Human Umbilical Vein Endothelial Cells by Targeting the PI3K/Akt/mTOR Pathway,” Molecular Medicine Reports 18, no. 3 (2018): 2798–2806. 30015881 10.3892/mmr.2018.9236 PMC6102700

[120] J. Lv , L. Yang , R. Guo , et al., “Ox‐LDL‐Induced microRNA‐155 Promotes Autophagy in Human Endothelial Cells via Repressing the Rheb/mTOR Pathway,” Cellular Physiology and Biochemistry 43, no. 4 (2017): 1436–1448. 29017169 10.1159/000481875

[121] C. Wang , W. Yang , X. Liang , et al., “MicroRNA‐761 Modulates Foam Cell Formation and Inflammation Through Autophagy in the Progression of Atherosclerosis,” Molecular and Cellular Biochemistry 474 (2020): 135–146. 32772311 10.1007/s11010-020-03839-y

[122] Z. Zhu , J. Li , R. Tong , X. Zhang , and B. Yu , “miR‐149 Alleviates Ox‐LDL‐Induced Endothelial Cell Injury by Promoting Autophagy Through Akt/mTOR Pathway,” Cardiology Research and Practice 2021, no. 1 (2021): 9963258. 34484820 10.1155/2021/9963258 PMC8416406

[123] D. Heras‐Sandoval , J. M. Pérez‐Rojas , J. Hernández‐Damián , and J. Pedraza‐Chaverri , “The Role of PI3K/AKT/mTOR Pathway in the Modulation of Autophagy and the Clearance of Protein Aggregates in Neurodegeneration,” Cellular Signalling 26, no. 12 (2014): 2694–2701. 25173700 10.1016/j.cellsig.2014.08.019

[124] X. Li , X. Hu , J. Wang , et al., “Inhibition of Autophagy via Activation of PI3K/Akt/mTOR Pathway Contributes to the Protection of Hesperidin Against Myocardial Ischemia/Reperfusion Injury,” International Journal of Molecular Medicine 42, no. 4 (2018): 1917–1924. 30066841 10.3892/ijmm.2018.3794 PMC6108872

[125] P. Liang , B. Jiang , Y. Li , et al., “Autophagy Promotes Angiogenesis via AMPK/Akt/mTOR Signaling During the Recovery of Heat‐Denatured Endothelial Cells,” Cell Death & Disease 9, no. 12 (2018): 1152. 30455420 10.1038/s41419-018-1194-5 PMC6242874

[126] B. J. Wang , W. L. Zheng , N. N. Feng , et al., “The Effects of Autophagy and PI3K/AKT/m‐TOR Signaling Pathway on the Cell‐Cycle Arrest of Rats Primary Sertoli Cells Induced by Zearalenone,” Toxins 10, no. 10 (2018): 398. 30274213 10.3390/toxins10100398 PMC6215106

[127] Y. Pu , Y. Zhou , T. Guo , et al., “MIF Promotes Phenotypic Switching of VSMCs via AKT/mTOR‐Mediated Autophagy Regulation in Aortic Dissection,” FASEB Journal 39, no. 19 (2025): e71079. 41001812 10.1096/fj.202501761R

[128] Z. Zhu , C. Yang , A. Iyaswamy , et al., “Balancing mTOR Signaling and Autophagy in the Treatment of Parkinson's Disease,” International Journal of Molecular Sciences 20, no. 3 (2019): 728. 30744070 10.3390/ijms20030728 PMC6387269

[129] W. Shao , S. Wang , X. Wang , et al., “miRNA‐29a Inhibits Atherosclerotic Plaque Formation by Mediating Macrophage Autophagy via PI3K/AKT/mTOR Pathway,” Aging (Albany NY) 14, no. 5 (2022): 2418. 35288486 10.18632/aging.203951 PMC8954956

[130] G. Wang , S. Y. Jing , G. Liu , et al., “miR‐99a‐5p: A Potential New Therapy for Atherosclerosis by Targeting mTOR and Then Inhibiting NLRP3 Inflammasome Activation and Promoting Macrophage Autophagy,” Disease Markers 2022 (2022): 7172583. 35968506 10.1155/2022/7172583 PMC9374553

[131] J. A. Deiuliis , “MicroRNAs as Regulators of Metabolic Disease: Pathophysiologic Significance and Emerging Role as Biomarkers and Therapeutics,” International Journal of Obesity 40, no. 1 (2016): 88–101. 26311337 10.1038/ijo.2015.170 PMC4722234

[132] Q. Jiang , Y. Li , Q. Wu , L. Huang , J. Xu , and Q. Zeng , “Pathogenic Role of microRNAs in Atherosclerotic Ischemic Stroke: Implications for Diagnosis and Therapy,” Genes & Diseases 9, no. 3 (2022): 682–696. 35782982 10.1016/j.gendis.2021.01.001 PMC9243347

[133] G. Luo , Z. Zhou , Z. Cao , et al., “M2 Macrophage‐Derived Exosomes Induce Angiogenesis and Increase Skin Flap Survival Through HIF1AN/HIF‐1α/VEGFA Control,” Archives of Biochemistry and Biophysics 751 (2024): 109822. 38030054 10.1016/j.abb.2023.109822

[134] Y. Wang , J. Zhang , Y. Gao , et al., “MiRNA‐27a‐5p Alleviates Diabetic Vascular Injury via Modulating Autophagy by Targeting NOX4,” Free Radical Biology and Medicine 241 (2025): 526–542. 41046944 10.1016/j.freeradbiomed.2025.09.057

[135] Z. Geng , F. Xu , and Y. Zhang , “MiR‐129‐5p‐Mediated Beclin‐1 Suppression Inhibits Endothelial Cell Autophagy in Atherosclerosis,” American Journal of Translational Research 8, no. 4 (2016): 1886. 27186312 PMC4859917

[136] M. Ouimet , H. Ediriweera , M. S. Afonso , et al., “microRNA‐33 Regulates Macrophage Autophagy in Atherosclerosis,” Arteriosclerosis, Thrombosis, and Vascular Biology 37, no. 6 (2017): 1058–1067. 28428217 10.1161/ATVBAHA.116.308916 PMC5494696

[137] J. Wang , W. N. Wang , S. B. Xu , et al., “Micro RNA‐214‐3p: A Link Between Autophagy and Endothelial Cell Dysfunction in Atherosclerosis,” Acta Physiologica 222, no. 3 (2018): e12973. 10.1111/apha.12973 28888077

[138] J. Wang , W. N. Wang , S. B. Xu , et al., “MicroRNA‐214‐3p: A Link Between Autophagy and Endothelial Cell Dysfunction in Atherosclerosis,” Acta Physiologica (Oxford, England) 222, no. 3 (2018): e12973. 10.1111/apha.12973 28888077

[139] S. Yin , S. Yang , X. Pan , et al., “MicroRNA‑155 Promotes ox‑LDL‑Induced Autophagy in Human Umbilical Vein Endothelial Cells by Targeting the PI3K/Akt/mTOR Pathway,” Molecular Medicine Reports 18, no. 3 (2018): 2798–2806. 30015881 10.3892/mmr.2018.9236 PMC6102700

[140] Z. Zhang , X. Pan , S. Yang , et al., “miR‐155 Promotes Ox‐LDL‐Induced Autophagy in Human Umbilical Vein Endothelial Cells,” Mediators of Inflammation 2017, no. 1 (2017): 9174801. 28659666 10.1155/2017/9174801 PMC5474275

[141] X. Zhang , L. Zai , Z. Tao , D. Wu , M. Lin , and J. Wan , “miR‐145‐5p Affects Autophagy by Targeting CaMKIIδ in Atherosclerosis,” International Journal of Cardiology 360 (2022): 68–75. 35597494 10.1016/j.ijcard.2022.05.039

[142] B. Wang , Y. Zhong , D. Huang , and J. Li , “Macrophage Autophagy Regulated by miR‐384‐5p‐Mediated Control of Beclin‐1 Plays a Role in the Development of Atherosclerosis,” American Journal of Translational Research 8, no. 2 (2016): 606–614. 27158352 PMC4846909

[143] J. R. Qi , D. R. Zhao , L. Zhao , F. Luo , and M. Yang , “MiR‐520a‐3p Inhibited Macrophage Polarization and Promoted the Development of Atherosclerosis via Targeting UVRAG in Apolipoprotein E Knockout Mice,” Frontiers in Molecular Biosciences 7 (2020): 621324. 33768113 10.3389/fmolb.2020.621324 PMC7985160

[144] P. Ge , M. Gao , J. Du , J. Yu , and L. Zhang , “Downregulation of microRNA‐512‐3p Enhances the Viability and Suppresses the Apoptosis of Vascular Endothelial Cells, Alleviates Autophagy and Endoplasmic Reticulum Stress as Well as Represses Atherosclerotic Lesions in Atherosclerosis by Adjusting Spliced/Unspliced Ratio of X‐Box Binding Protein 1 (XBP‐1S/XBP‐1U),” Bioengineered 12, no. 2 (2021): 12469–12481. 34783632 10.1080/21655979.2021.2006862 PMC8810154

[145] R. Menghini , V. Casagrande , A. Marino , et al., “MiR‐216a: A Link Between Endothelial Dysfunction and Autophagy,” Cell Death and Disease 5, no. 1 (2014): e1029. 24481443 10.1038/cddis.2013.556 PMC4040670

[146] B. W. Wu , Y. Liu , M. S. Wu , et al., “Downregulation of microRNA‐135b Promotes Atherosclerotic Plaque Stabilization in Atherosclerotic Mice by Upregulating Erythropoietin Receptor,” IUBMB Life 72, no. 2 (2020): 198–213. 31444954 10.1002/iub.2155

[147] X. Qin and J. Guo , “MicroRNA‐328‐3p Protects Vascular Endothelial Cells Against Oxidized Low‐Density Lipoprotein Induced Injury via Targeting Forkhead Box Protein O4 (FOXO4) in Atherosclerosis,” Medical Science Monitor 26 (2020): e921877. 32329448 10.12659/MSM.921877 PMC7195608

[148] H. Zhu , H. Liang , Z. Gao , et al., “MiR‐483‐5p Downregulation Alleviates Ox‐LDL Induced Endothelial Cell Injury in Atherosclerosis,” BMC Cardiovascular Disorders 23, no. 1 (2023): 521. 37891465 10.1186/s12872-023-03496-1 PMC10612234

[149] T. Sun , M. Y. Li , P. F. Li , and J. M. Cao , “MicroRNAs in Cardiac Autophagy: Small Molecules and Big Role,” Cells 7, no. 8 (2018): 104. 30103495 10.3390/cells7080104 PMC6116024

[150] M. Zou , F. Wang , R. Gao , et al., “Autophagy Inhibition of Hsa‐miR‐19a‐3p/19b‐3p by Targeting TGF‐β R II During TGF‐β1‐Induced Fibrogenesis in Human Cardiac Fibroblasts,” Scientific Reports 6 (2016): 24747. 27098600 10.1038/srep24747 PMC4838850

[151] X. D. Zhao , R. H. Qin , J. J. Yang , et al., “DNMT3A Controls miR‐200b in Cardiac Fibroblast Autophagy and Cardiac Fibrosis,” Inflammation Research 67, no. 8 (2018): 681–690. 29786779 10.1007/s00011-018-1159-2

[152] Z. M. Zhang , R. Lu , P. Wang , et al., “Structural Basis for DNMT3A‐Mediated De Novo DNA Methylation,” Nature 554, no. 7692 (2018): 387–391. 29414941 10.1038/nature25477 PMC5814352

[153] M. Shumliakivska , G. Luxán , I. Hemmerling , et al., “DNMT3A Clonal Hematopoiesis‐Driver Mutations Induce Cardiac Fibrosis by Paracrine Activation of Fibroblasts,” Nature Communications 15, no. 1 (2024): 606. 10.1038/s41467-023-43003-w PMC10799021 38242884

[154] M. Yang , J. J. Song , X. C. Yang , G. Z. Zhong , and J. C. Zhong , “MiRNA‐122‐5p Inhibitor Abolishes Angiotensin II‐Mediated Loss of Autophagy and Promotion of Apoptosis in Rat Cardiofibroblasts by Modulation of the Apelin‐AMPK‐mTOR Signaling,” In Vitro Cellular & Developmental Biology. Animal 58, no. 2 (2022): 136–148. 35133561 10.1007/s11626-022-00651-4

[155] C. J. Oldfield , T. A. Duhamel , and N. S. Dhalla , “Mechanisms for the Transition From Physiological to Pathological Cardiac Hypertrophy,” Canadian Journal of Physiology and Pharmacology 98, no. 2 (2020): 74–84. 31815523 10.1139/cjpp-2019-0566

[156] M. Al Hemairy , S. Amin , M. Hijji , M. Serhani , and M. Al Ahmad , “Integrated and Scalable Architecture for Providing Cost‐Effective Remote Health Monitoring,” (2016), Paper presented at: 2016 9th International Conference on Developments in eSystems Engineering (DeSE).

[157] L. Jin , Y. Zhou , L. Han , and J. Piao , “MicroRNA302‐367‐PI3K‐PTEN‐AKT‐mTORC1 Pathway Promotes the Development of Cardiac Hypertrophy Through Controlling Autophagy,” In Vitro Cellular & Developmental Biology. Animal 56, no. 2 (2020): 112–119. 31845077 10.1007/s11626-019-00417-5

[158] X. Xu , Y. L. Su , J. Y. Shi , Q. Lu , and C. Chen , “MicroRNA‐17‐5p Promotes Cardiac Hypertrophy by Targeting Mfn2 to Inhibit Autophagy,” Cardiovascular Toxicology 21, no. 9 (2021): 759–771. 34120306 10.1007/s12012-021-09667-w

[159] S. Liu , L. Su , J. Li , et al., “Inhibition of miR‐146b‐5p Alleviates Isoprenaline‐Induced Cardiac Hypertrophy via Regulating DFCP1,” Molecular and Cellular Endocrinology 589 (2024): 112252. 38649132 10.1016/j.mce.2024.112252

[160] C. Zhang , K. Zhang , F. Huang , et al., “Exosomes, the Message Transporters in Vascular Calcification,” Journal of Cellular and Molecular Medicine 22, no. 9 (2018): 4024–4033. 29892998 10.1111/jcmm.13692 PMC6111818

[161] X. Wen , P. Shang , H. Chen , et al., “Evolutionary Study and Structural Basis of Proton Sensing by Mus GPR4 and Xenopus GPR4,” Cell 188, no. 3 (2025): 653–670.e624. 39753131 10.1016/j.cell.2024.12.001

[162] F.‐X.‐Z. Li , J.‐J. Liu , F. Xu , et al., “Cold Exposure Protects Against Medial Arterial Calcification Development via Autophagy,” Journal of Nanobiotechnology 21, no. 1 (2023): 226. 37461031 10.1186/s12951-023-01985-1 PMC10351118

[163] T. H. Xu , X. B. Qiu , Z. T. Sheng , et al., “Restoration of microRNA‐30b Expression Alleviates Vascular Calcification Through the mTOR Signaling Pathway and Autophagy,” Journal of Cellular Physiology 234, no. 8 (2019): 14306–14318. 30701530 10.1002/jcp.28130 PMC13482998

[164] J. Cao , C. Chen , Q. Chen , et al., “Extracellular Vesicle miR‐32 Derived From Macrophage Promotes Arterial Calcification in Mice With Type 2 Diabetes via Inhibiting VSMC Autophagy,” Journal of Translational Medicine 20, no. 1 (2022): 307. 35794619 10.1186/s12967-022-03502-8 PMC9258116

[165] A. Goliopoulou , E. Oikonomou , A. Antonopoulos , et al., “Expression of Tissue microRNAs in Ascending Aortic Aneurysms and Dissections,” Angiology 74, no. 1 (2023): 88–94. 35503041 10.1177/00033197221098295

[166] Z. Zhong , J. Wu , K. Yuan , et al., “Upregulation of microRNA‐205 Is a Potential Biomarker for Intracranial Aneurysms,” Neuroreport 30, no. 12 (2019): 812–816. 31283712 10.1097/WNR.0000000000001279

[167] Q. Yu , Q. Li , X. Yang , et al., “Dexmedetomidine Suppresses the Development of Abdominal Aortic Aneurysm by Downregulating the mircoRNA‐21/PDCD 4 Axis,” International Journal of Molecular Medicine 47, no. 5 (2021): 90. 33786608 10.3892/ijmm.2021.4923 PMC8029612

[168] J. Peng , X. He , L. Zhang , and P. Liu , “MicroRNA‐26a Protects Vascular Smooth Muscle Cells Against H^2^O^2^‐Induced Injury Through Activation of the PTEN/AKT/mTOR Pathway,” International Journal of Molecular Medicine 42, no. 3 (2018): 1367–1378. 29956734 10.3892/ijmm.2018.3746 PMC6089772

[169] M. Zhang and Z. Wang , “Downregulation of miR143/145 Gene Cluster Expression Promotes the Aortic Media Degeneration Process via the TGF‐β1 Signaling Pathway,” American Journal of Translational Research 11, no. 1 (2019): 370–378. 30787994 PMC6357338

[170] Y. Yang , K. Yamagishi , T. Kihara , et al., “Smoking Cessation and Mortality From Aortic Dissection and Aneurysm: Findings From the Japan Collaborative Cohort (JACC) Study,” Journal of Atherosclerosis and Thrombosis 30, no. 4 (2023): 348–363. 35718450 10.5551/jat.63258 PMC10067343

[171] A. J. Doyle , E. M. Redmond , D. L. Gillespie , et al., “Differential Expression of Hedgehog/Notch and Transforming Growth Factor‐β in Human Abdominal Aortic Aneurysms,” Journal of Vascular Surgery 62, no. 2 (2015): 464–470. 24768363 10.1016/j.jvs.2014.02.053 PMC4208950

[172] W. H. Wang , Y. H. Wang , L. L. Zheng , X. W. Li , F. Hao , and D. Guo , “MicroRNA‐29a: A Potential Biomarker in the Development of Intracranial Aneurysm,” Journal of the Neurological Sciences 364 (2016): 84–89. 27084222 10.1016/j.jns.2016.03.010

[173] W. Zhao , H. Zhang , and J. Y. Su , “MicroRNA‐29a Contributes to Intracranial Aneurysm by Regulating the Mitochondrial Apoptotic Pathway,” Molecular Medicine Reports 18, no. 3 (2018): 2945–2954. 30015903 10.3892/mmr.2018.9257

[174] Z. Zheng , Y. Chen , Y. Wang , Y. Li , and Q. Cheng , “MicroRNA‐513b‐5p Targets COL1A1 and COL1A2 Associated With the Formation and Rupture of Intracranial Aneurysm,” Scientific Reports 11, no. 1 (2021): 14897. 34290266 10.1038/s41598-021-94116-5 PMC8295310

[175] J. Wu , I. Gareev , O. Beylerli , et al., “Circulating miR‐126 as a Potential Non‐Invasive Biomarker for Intracranial Aneurysmal Rupture: A Pilot Study,” Current Neurovascular Research 18, no. 5 (2021): 525–534. 34923944 10.2174/1567202619666211217142116

[176] F. Yang , W. W. Xing , D. W. Shen , M. F. Tong , and F. M. Xie , “Effect of miR‐126 on Intracranial Aneurysms and Its Predictive Value for Rupture of Aneurysms,” European Review for Medical and Pharmacological Sciences 24, no. 6 (2020): 3245–3253. 32271441 10.26355/eurrev_202003_20691

[177] F. Piccirillo , M. Carpenito , G. Verolino , et al., “Changes of the Coronary Arteries and Cardiac Microvasculature With Aging: Implications for Translational Research and Clinical Practice,” Mechanisms of Ageing and Development 184 (2019): 111161. 31647940 10.1016/j.mad.2019.111161

[178] M. H. Bao , Q. L. Lv , H. G. Li , Y. W. Zhang , B. F. Xu , and B. S. He , “A Novel Putative Role of TNK1 in Atherosclerotic Inflammation Implicating the Tyk2/STAT1 Pathway,” Mediators of Inflammation 2020 (2020): 6268514. 32694928 10.1155/2020/6268514 PMC7368939

[179] M. Fitzpatrick , W. Wood, 3rd , S. De , et al., “Age‐Related Changes in microRNA Levels in Serum,” Aging (Albany NY) 5, no. 10 (2013): 725–740. 24088671 10.18632/aging.100603 PMC3838776

[180] W. E. Hughes , A. M. Beyer , and D. D. Gutterman , “Vascular Autophagy in Health and Disease,” Basic Research in Cardiology 115, no. 4 (2020): 41. 32506214 10.1007/s00395-020-0802-6 PMC12306491

[181] X. Zhang , G. Azhar , and J. Y. Wei , “The Expression of microRNA and microRNA Clusters in the Aging Heart,” PLoS One 7, no. 4 (2012): e34688. 22529925 10.1371/journal.pone.0034688 PMC3329493

[182] J. Yuan , H. Chen , D. Ge , et al., “Mir‐21 Promotes Cardiac Fibrosis After Myocardial Infarction via Targeting Smad7,” Cellular Physiology and Biochemistry 42, no. 6 (2017): 2207–2219. 28817807 10.1159/000479995

[183] X. Zhou , H. Xu , Z. Liu , et al., “miR‐21 Promotes Cardiac Fibroblast‐To‐Myofibroblast Transformation and Myocardial Fibrosis by Targeting Jagged1,” Journal of Cellular and Molecular Medicine 22, no. 8 (2018): 3816–3824. 29808534 10.1111/jcmm.13654 PMC6050485

[184] R. A. Boon , K. Iekushi , S. Lechner , et al., “MicroRNA‐34a Regulates Cardiac Ageing and Function,” Nature 495, no. 7439 (2013): 107–110. 23426265 10.1038/nature11919

[185] G. C. van Almen , W. Verhesen , R. E. van Leeuwen , et al., “MicroRNA‐18 and microRNA‐19 Regulate CTGF and TSP‐1 Expression in Age‐Related Heart Failure,” Aging Cell 10, no. 5 (2011): 769–779. 21501375 10.1111/j.1474-9726.2011.00714.x PMC3193380

[186] B. W. van Balkom , O. G. De Jong , M. Smits , et al., “Endothelial Cells Require miR‐214 to Secrete Exosomes That Suppress Senescence and Induce Angiogenesis in Human and Mouse Endothelial Cells,” Blood, The Journal of the American Society of Hematology 121, no. 19 (2013): 3997–4006. 10.1182/blood-2013-02-478925 23532734

